# Supplementary material for: Patterns and determinants of the global herbivorous mycobiome
Source: Nat Commun. 2023 Jun 26;14:3798. doi: 10.1038/s41467-023-39508-z (PMC10293281; doi:10.1038/s41467-023-39508-z)
Supplement: Supplementary file 1 — Supplementary Information [file 41467_2023_39508_MOESM1_ESM.pdf]

Supplementary Information: Patterns and determinants of the global herbivorous mycobiome

Casey H. Meili[1], Adrienne L. Jones[1], Alex Arreola[1], Jeff Habel[1], Carrie J. Pratt[1], Radwa A. Hanafy[1], Yan Wang[2], Aymen S. Yassin[3], Moustafa A. TagElDein[3], Christina D. Moon[4], Peter H. Janssen[4], Mitesh Shrestha[5], Prajwal Rajbhandari[5], Magdalena Nagler[6], Julia M. Vinzelj[6], Sabine M. Podmirseg[6], Jason E. Stajich[7], Arthur L. Goetsch[8], Jerry Hayes[8], Diana Young[9], Katerina Fliegerova[10], Diego Javier Grilli[11], Roman Vodička[12], Giuseppe Moniello[13], Silvana Mattiello[14], Mona T. Kashef[2], Yosra I. Nagy[2], Joan A. Edwards[15], Sumit Singh Dagar[16], Andrew P. Foote[17], Noha H. Youssef[1*,] Mostafa S. Elshahed[1*]

[1] Oklahoma State University, Department of Microbiology and Molecular Genetics, Stillwater, Oklahoma, USA.
[2]Department of Biological Sciences, University of Toronto Scarborough, Toronto, Ontario, Canada.
[3] Department of Microbiology and Immunology, Faculty of Pharmacy, Cairo University, Cairo, Egypt.
[4]AgResearch, Grasslands Research Centre, Palmerston North, New Zealand.
[5]Department of Applied Microbiology and Food Technology, Research Institute for Bioscience and Biotechnology (RIBB), Kathmandu, Nepal.
[6]Universität Innsbruck, Faculty of Biology, Department of Microbiology, Innsbruck, Austria.
[7]Department of Microbiology and Plant Pathology, University of California, Riverside, Riverside, CA, USA
[8]Langston University, Langston, Oklahoma, USA.
[9]Bavarian State Center for Agriculture, Freising, Germany.
[10]Institute of Animal Physiology and Genetics Czech Academy of Sciences, Prague, Czechia.
[11]Área de Microbiología, Facultad de Ciencias Médicas, Universidad Nacional de Cuyo, Mendoza, Argentina
[12]Prague Zoo, Prague, Czechia.
[13]Department of Veterinary Medicine, University of Sassari, Sardinia, Italy.
[14]University of Milan, Dept. of Agricultural and Environmental Sciences, Milan, Italy.
[15] Anaerobic Fungi Network, Kerkdriel, Netherlands
[16]Bioenergy Group, Agharkar Research Institute, Pune, India.
[17]Department of Animal and Food Sciences, Oklahoma State University, Stillwater, Oklahoma, USA.

*Corresponding author: Address 1110 S. Innovation way, Stillwater, OK. Email: Mostafa@Okstate.edu and Noha@Okstate.edu

I. Supplementary methods

Sampling. A total of 661 samples belonging to thirty-four different mammalian animal species and nine families of foregut ruminant, foregut pseudoruminant, and hindgut fermenters were included in the final analysis (Figures 1a-b, Table S2). Samples were obtained from 15 different laboratories using a standardized procedure. Fresh, moist fecal samples originating from a single animal were scooped immediately post-defecation into sterile 50-ml plastic tubes and rapidly sealed. Samples were transferred to the laboratory on ice, usually within one hour, and stored at -20ºC prior to DNA extraction. All individuals collecting samples from domesticated animals were owners or caretakers and were authorized and trained in animal keeping and husbandry. For domesticated animals reared in a research institution, all IRB protocols for animal rearing were observed. Sampling wild herbivores was conducted through a partnership with hunting communities, alleviating the need for obtaining hunting permits. All hunters had the appropriate licenses, and the animals were shot on public land during the hunting season.

DNA extraction. DNA extractions were conducted in eight laboratories (Oklahoma, USA: 418 samples, Egypt: 74 samples, Nepal: 25 samples, Austria: 10 samples, Italy:38 Samples, Czechia: 24 samples, Germany: 31 samples, and New Zealand: 35 samples) and followed the manufacturer's instructions for DNeasy Plant Pro Kit (Qiagen®, Germantown, Maryland). Few samples were from a prior study [1]. The kit was shipped from the USA to labs in Nepal and Egypt and was purchased independently elsewhere.

*PCR amplification, and Illumina sequencing.*The 28S rRNA was used as the phylomarker for this diversity survey since as previously suggested for AGF genus-level delineation [2, 3]. The LSU rRNA has been regarded as a more reliable marker for AGF diversity characterization (compared to the general fungal phylomarker ITS1 region) by the scientific community [4], and has been

previously employed in AGF culture-independent diversity surveys [5]. By comparison, the ITS1 region suffers from high within-strain sequence divergence, and length variability [5, 6]. All PCR amplifications were conducted in a single laboratory to eliminate inter-laboratory variability. All reactions utilized the DreamTaq Green PCR Master Mix (ThermoFisher, Waltham, Massachusetts), and primers targeting the D2 region of the LSU rRNA (AGF-LSU-EnvS For: 5'-GCGTTTRRCACCASTGTTGTT-3', AGF-LSU-EnvS Rev: 5'-GTCAACATCCTAAGYGTAGGTA-3') for amplification. The primers were recently developed and extensively evaluated for sensitivity, specificity, Neocallimastigomycota coverage, and ability to differentiate between all known AGF genera and candidate genera [7]. The primers target a ~370 bp region of the LSU rRNA gene (corresponding to the D2 domain), hence allowing for high throughput sequencing using the Illumina MiSeq platform. Primers were modified to include the Illumina overhang adaptors. PCR reactions contained 2 µl of DNA, 25 µl of the DreamTaq 2X master mix (Life Technologies, Carlsbad, California), and 2 µl of each primer (10 µM) in a 50 µl reaction mix. The PCR protocol consisted of an initial denaturation for 5 min at 95 °C followed by 40 cycles of denaturation at 95 °C for 1 min, annealing at 55 °C for 1 min and elongation at 72 °C for 1 min, and a final extension of 72 °C for 10 min. PCR products were individually cleaned to remove unannealed primers using PureLink® gel extraction kit (Life Technologies, Carlsbad, California), and the clean product was used in a second PCR reaction to attach the dual indices and Illumina sequencing adapters using Nexterra XT index kit v2 (Illumina Inc., San Diego, California). These second PCR products were then cleaned using PureLink® gel extraction kit (Life Technologies, Carlsbad, California), individually quantified using Qubit® (Life Technologies, Carlsbad, California), and pooled using the Illumina library pooling calculator (https://support.illumina.com/help/pooling-calculator/pooling-calculator.htm)

to prepare 4-5 nM libraries. Pooled libraries (300-350 samples) were sequenced at the University of Oklahoma Clinical Genomics Facility using the MiSeq platform.

Complementary PacBio sequencing. The 700-750 bp D1/D2 LSU fragment has recently been adopted [4] as the gold standard for AGF genus- and species-level assignments, as well as for circumscribing boundaries between various AGF clades. However, the length of the region precludes the use of Illumina technology for high throughput sequencing and necessitates the use of the technically more cumbersome and expensive Sanger or PacBio sequencing platforms. Therefore, as a complementary approach to Illumina sequencing, we conducted PacBio sequencing on a subset of the Illumina-sequenced samples (n=61) with the following goals in mind: (1) to ensure that community membership, structure, and diversity estimates obtained with the shorter fragment (D2 region only) are comparable to those obtained with the previously utilized D1/D2 region, (2) to ensure the feasibility and resolution power of the D2 region in identifying and differentiating various fungal lineages, and (3) to confirm the presence of the unexpectedly high number of putative novel genera, provide full-length representative sequences [4], and amend the curated D1/D2 LSU rRNA database currently handled by the authors as part of the broader AGF community of researchers (www.anaerobicfungi.org). For amplification of the D1/D2 LSU region, we paired a universal fungal forward primer (NL1: 5'-GCATATCAATAAGCGGAGGAAAAG-3') with an AGF-specific reverse primer (GG-NL4: 5'-TCAACATCCTAAGCGTAGGTA-3') [5, 8]. Primers were barcoded to allow multiplexing and PacBio sequencing. The PCR protocol consisted of an initial denaturation for 5 min at 95°C followed by 40 cycles of denaturation at 95°C for 1 min, annealing at 55°C for 1 min and elongation at 72°C for 1 min, and a final extension of 72°C for 10 min. Amplicons were purified using PureLink® gel extraction kit (Life Technologies), quantified using Qubit® (Life

Technologies), pooled, and sequenced at Washington State University core facility using one cell of the SMRT Pacific Biosciences (PacBio) Sequel II system.

Illumina and PacBio sequence processing. Forward and reverse Illumina reads were assembled using make.contigs command in mothur [9], followed by screening to remove sequences with ambiguous bases, sequences with homopolymer stretches longer than 8 bases, and sequences that were shorter than 200 or longer than 380 bp. For PacBio sequences, raw reads were processed using the official PacBio pipeline (RS_Subreads.1) (http://files.pacb.com/software/smrtanalysis/2.2.0/doc/smrtportal/help/!SSL!/Webhelp/CS_Prot_RS_Subreads.htm), and filtered using default settings of the minimum read length, and minimum read quality. Remaining reads were then processed with the PacBio RS_ReadsOfInsert protocol (http://files.pacb.com/software/smrtanalysis/2.2.0/doc/smrtportal/help/!SSL!/Webhelp/CS_Prot_RS_ReadsOfInsert.htm) for generating single-molecule consensus reads from the insert template. Circular consensus sequences were further processed in mothur [9] to remove any sequence with an average quality score < 25, sequences with ambiguous bases, sequences not containing the correct barcode, sequences with more than a 2 bp difference in the primer sequence, and/or sequences with homopolymer stretches longer than 8 bp. To identify any CCS with the primer sequence in the middle, we performed a standalone blastn-short using the primer sequence as the query and removed the identified sequences using the remove.seqs command in mothur.

Taxonomic and phylogenetic assignments. To examine how taxa delineation cutoffs previously proposed based on the D1/D2 region [4, 5] correlate to those of the shorter Illumina-generated D2 LSU fragments obtained in this study, we conducted preliminary comparison of all possible pairwise sequence divergence values from the alignment of the whole D1/D2 region of 206 reference sequences (available at www.anaerobicfungi.org), to those from the truncated

alignment covering the D2 region only (corresponding to the region that would be amplified using the AGF-LSU-EnvS primer pair above). Sequence divergence estimates from the two sets of alignments were very well correlated (R^2= 0.885, Figure S9). However, comparison of pairwise sequence divergence using the whole D1/D2 region versus the D2 region suggests that the 2% sequence divergence cutoff previously proposed as the threshold for delineating AGF species using the D1/D2 region (based on comparisons of validly described species) [4] is equivalent to 3.5% using the D2 region only, and the 3% sequence divergence cutoff previously proposed as the threshold for delineating AGF genera using the D1/D2 region [4] is equivalent to 5.1% using the D2 region only (Figure S9).

Therefore, pairwise distances were used to cluster the sequences into species-level OTUs using the proposed sequence threshold of 3.5%. On the other hand, prior research has shown that using specific thresholds for genus-level delineation in AGF is problematic. For example, some genera were found to harbor higher intra-genus D1/D2-LSU region sequence divergence values (e.g. the genus *Piromyces* intra-genus D1/D2-LSU region sequence divergence ranges between 0% and 5.7%), while others diverge by <2% from neighboring genera (e.g. the *Anaeromyces-Liebetanzomyces-Capellomyces-Oontomyces* clade harbors inter-genus D1/D2-LSU region sequence divergence values ranging between 1.8% and 2.5%) [4]. Therefore, we refrained from using a predetermined threshold to assign sequences to AGF genera and instead used a two-tier approach for genus-level phylogenetic placement. First, sequences were compared by blastn to the curated D1/D2 LSU rRNA AGF database (www.anaerobicfungi.org), and sequences were classified as their first hit taxonomy if the percentage similarity to the first hit was >96% and the two sequences were aligned over >70% of the query sequence length. For all sequences that could not be confidently assigned to an AGF genus by blastn, insertion into a reference LSU tree

(with representatives from all cultured and uncultured AGF genera and candidate genera) was used to assess novelty. Briefly, unaffiliated sequences (100-200 at a time) were aligned to the reference database using align.seqs in mothur, and the alignment was used to construct maximum likelihood phylogenetic trees in FastTree [10] using the GTR model. Sequences were assigned to a novel genus when they cluster as an independent genus-level clade with high (>70%) bootstrap support in the ML tree. Representatives of novel genera were sequentially added to the reference LSU tree, before processing the next batch of unaffiliated sequences. Intra-genus D2 region sequence divergence for sequences assigned to any novel genus never exceeded 5% (equivalent to 3% D1/D2 sequence divergence). Following the assignment of all sequences to either an existing or a novel genus, final trees with 5-10 representatives of each genus were generated in IQ-TREE [11] using the alignment of the D2 region. ModelFinder [12] through IQ-TREE was used to select the best substitution model (with the lowest Bayesian information criterion). Maximum likelihood trees were constructed under the predicted best model, with the -alrt 1000, the -bb 1000, and the --abayes options added to the commandline for performing the Shimodaira–Hasegawa approximate likelihood ratio test (SH-aLRT), the ultrafast bootstrap (UFB) [13], and the approximate Bayes test. This resulted in the generation of phylogenetic trees with three support values (SH-aLRT, aBayes, and UFB) on each branch. Phylogenetic analysis as described above resulted in the confident assignment of every single sequence to either an existing cultured, or uncultured genus, or to a novel genus. These genus-level assignments were then used to build a taxonomy file in mothur, which was subsequently used to build a shared file using the mothur commands phylotype and make.shared.

Amplicon sequence variants (ASVs) have recently been gaining popularity and momentum in describing diversity in bacterial [14, 15], archaeal [16], and fungal [17] surveys, a

proposition augmented by improved sequence quality and stringent quality control procedures on all sequencing platforms [18, 19, 20, 21], we, however, refrained from using ASVs in this study, due to the fact that a significant level of within-strain divergence (ranging between 0.1%-1.9% [5]) is observed in the multiple LSU rRNA gene copies (estimated around 170 per genome). As such, use of ASVs, with its emphasis on exact sequencing identity [18, 19, 20] would immensely overestimate AGF diversity and bias community structure estimates.

Role of stochastic versus deterministic processes in shaping AGF community assembly. We assessed the contribution of various deterministic and stochastic processes to the AGF community assembly using both normalized stochasticity ratio (NST) [22], and the null-model-based quantitative framework implemented by [23, 24]. The NST index infers ecological stochasticity, however, values do not pinpoint the sources of selection (determinism) or stochasticity. Also, NST values are calculated solely based on taxonomic diversity indices with no consideration to the phylogenetic turnover in the community. To quantify the contribution of various deterministic (homogenous and heterogenous selection) and stochastic (dispersal preference, limitation, drift) processes in shaping the AGF community assembly, we used a two-step null-model-based quantitative framework that makes use of both taxonomic (RC_{Bray}) and phylogenetic (β-NRI) β-diversity metrics [23, 24]. The NST package in R was used to calculate the normalized stochasticity ratio (NST) based on two taxonomic β-diversity dissimilarity metrics; the incidence-based Jaccard index, and the abundance-based Bray-Curtis index, where an NST value of >50% indicates a more stochastic assembly, while values <50% indicate a more deterministic assembly. To test the significance of difference between pairs of animal species (for animals with more than 20 individuals; cows, goats, sheep, deer, and horses), animal families (for families with more than 10 individuals; Bovidae, Cervidae, Camelidae, Equidae,

and Elephantidae), and animal gut types (foregut, pseudoruminant, and hindgut), we used the function nst.boot in the NST package in R to randomly draw samples within each group followed by bootstrapping of NST values. Obtained values were then compared using Wilcoxon test with Benjamini-Hochberg adjustment. The iCAMP R package was used to calculate values of beta net relatedness index (β-NRI) and modified Raup-Crick metric based on Bray Curtis metric (RC_{Bray}) using the function bNRIn.p to evaluate the turnover for both phylogenetic, and taxonomic diversity. Values of β-NRI were used first to partition selective processes into homogenous (number of pairwise comparisons with β-NRI values <-2), and heterogenous selection (number of pairwise comparisons with β-NRI values >2). All other pairwise comparisons (with absolute β-NRI values <2) are considered as contributing to stochastic processes (not assigned to selection), and can be further broken down into dispersal and drift based on the taxonomic diversity (values of RC_{Bray}). Specifically for these, the number of pairwise comparisons with absolute values of RC_{Bray} <0.95 are considered as contributing to drift, while the number of pairwise comparisons with absolute values of RC_{Bray} >0.95 are considered contributing to dispersal. This last fraction can be further broken down into homogenizing dispersal (RC_{Bray} values <-0.95), and dispersal limitation (RC_{Bray} values >0.95). The contribution of each of these processes (homogenous selection, heterogenous selection, homogenizing dispersal, dispersal limitation, and drift) to the total AGF community assembly was calculated from the corresponding number of pairwise comparisons falling into each category as a percentage of all pairwise comparisons.

Factors impacting AGF diversity and community structure. We considered two types of factors that could potentially impact AGF diversity and community structure: host-associated factors, and non-host-associated factors. For host-associated factors, we considered animal species, animal family, and animal gut type, while for non-host-associated factors, we considered

animal domestication status, biogeography (country of origin), animal age, and animal sex. For testing the effect of biogeography, age, and sex on alpha diversity measures and community structure, we opted to carry out comparisons only on samples belonging to the same animal species in an attempt to control for other host-associated factors that might conflate the results. For these comparisons, only the four most-sampled animal species (cattle, goats, sheep, and horses) were considered.

Alpha diversity *measures.* Alpha diversity estimates (observed number of genera, Shannon, Simpson, and Inverse Simpson diversity indices) were calculated using the command estimate_richness in the phyloseq R package. For comparison of alpha diversity between samples, patterns were assessed in samples with at least 1000 sequences (n=421 samples) using the four indices, and two sampling strategies (with and without random subsampling of 1000 sequences) for eight total comparisons. The importance of various factors (host-associated factors, e.g., gut type, animal family, or animal species; domestication status, and biogeography) in shaping the observed patterns of alpha diversity was examined using ANOVA (calculated using the aov command in R). Only samples that have at least 10 replicates (at any of these host factor levels) were included in the analysis. These included foregut and hindgut (for the gut type factor comparison), families Bovidae, Cervidae, and Equidae (for the animal family comparison), cows, goats, sheep, deer, and horses (for the animal genus comparison), and domesticated and non-domesticated (for domestication status comparison). Additionally, post hoc Tukey HSD tests for multiple comparisons of means were run on the results of ANOVA (using TukeyHSD command in R) for all possible pairwise comparisons to identify the pairs of groups that are significantly different for each host factor.

As mentioned above, we opted to carry out comparisons of the effect of biogeography,

age, and sex only on samples belonging to the same animal species (only the four most-sampled animals were included) in an attempt to control for other host-associated factors that might conflate the results. Biogeography comparisons were conducted on cattle, goat, sheep, and horse datasets originating from the USA, Egypt, Germany, Italy, Austria, Czech Republic, New Zealand, and Argentina. ANOVA (calculated using the aov command in R) was used to identify the animal species whose AGF alpha diversity significantly differed between countries. For these animal datasets, post hoc Tukey HSD tests for multiple comparisons of means were run on the results of ANOVA (using TukeyHSD command in R) for all possible pairwise country comparisons to identify the pairs that are significantly different for each animal genus. Additionally, the effect of the US state of origin on AGF alpha diversity in cattle and horses was also tested using ANOVA followed by post hoc Tukey HSD tests for multiple comparisons of means for all possible pairwise state comparisons to identify the pairs that are significantly different for each animal species.

Age (young, <1 year; adult, >1 year), and sex (male versus female) comparisons were also considered only for the four most-sampled animals (cattle, goats, sheep, and horses). ANOVA followed by post hoc Tukey HSD tests were used.

AGF community structure. The genus-level shared file was used to calculate several beta diversity indices (including the phylogenetic similarity-based (e.g. unweighted and weighted Unifrac) using the ordinate command in the phyloseq R package. The pairwise values were used to construct ordination plots (both PCoA and NMDS) using the function plot_ordination in the phyloseq R package. RDA plots were also constructed using the genera abundance data following center log-ratio transformation (CLR). To assess the variability in community structure between samples belonging to each animal host species (only for animals with 4 or more

individuals), animal host family, animal gut type, and animal domestication status, we first calculated group centroids for each of these groups using the vegan command betadisper. Following, the ordination distance of each sample to its group centroid was calculated (as the Euclidean distance between two points), and distances from group centroids were plotted in a box and whisker plot (using the command boxplot in R). To partition the dissimilarity among the sources of variation (including animal host species, animal host family, animal gut type, and domestication status), PERMANOVA tests were run for each of the above beta diversity measures using the vegan command adonis, and the F-statistics p-values were compared to identify the host factors that significantly affect the AGF community structure. The percentage variance explained by each factor was calculated as the percentage of the sum of squares of each factor to the total sum of squares.

Due to the inherent sensitivity of PERMANOVA to the heterogeneity of variance among groups [25], and to further quantitatively assess factors that explain AGF diversity, we used three multivariate regression approaches based on matrices comparison: multiple regression of matrices (MRM), Mantel tests for matrices correlations, and Procrustes rotation. Bray-Curtis, and Jaccard dissimilarity matrices were first calculated from the genus shared file using vegdist command in vegan. Similarly, Unifrac weighted, and Unifrac unweighted dissimilarity matrices were calculated using the distance command in the phyloseq package. Each of these four AGF dissimilarity matrices were compared to a matrix of each of the host factors tested (animal host species, animal host family, animal gut type, and domestication status). For the animal host genus, a cophenetic matrix was calculated (using the command cophenetic in the ape R package) based on the newick tree downloaded from timetree.org and modified to include all the samples studied here with very short branch length between samples from the same animal species. For

the animal host family, animal gut type, and domestication status, since these were nominal values, matrices were constructed by Gower transformation [26]. Each of the AGF community dissimilarity matrices (n=4) was then correlated to each of the host factor matrices (n=4) using the commands MRM, and mantel in the ecodist R package, for running multiple regression on matrices, and Mantel tests, respectively. The Procrustes rotation was calculated using the protest command in the vegan R package. For each of the host factors tested, 12 total correlations (3 methods x 4 dissimilarity indices) were compared to evaluate the importance of the host factor in explaining the AGF community structure. This was achieved by comparing the p-values for significance of correlation, and coefficients (R^2 regression coefficients of the MRM analysis, Spearman correlation coefficients of the Mantel test, and symmetric orthogonal Procrustes statistic of the Procrustes analysis) for the importance of the factor in explaining community structure. Finally, to assess the sensitivity of multivariate regression methods to community composition variation among hosts of the same species, we permuted the MRM analysis 100 times, where one individual per animal species was randomly selected. For each of these permutations, and for each dissimilarity matrix-host factor comparison, a p-value and an R^2 regression coefficient were obtained. We considered a host factor significant in explaining AGF community structure, if in the permutation analysis, the p-value obtained was significant ($p < 0.05$) in at least 75 permutations.

To test for the effect of biogeography, sex, and age on the AGF community structure, and to overcome compounded effects from other host factors, we selected a subset of the whole dataset to include only samples from the four most-sampled animals (namely, cattle, goats, sheep, and horses). For each of these animal species, we calculated Bray-Curtis dissimilarity indices using the ordinate command in the phyloseq R package. To test for the significance of

the above three factors in describing AGF community structure in each animal genus, PERMANOVA tests were run using the vegan command adonis. The F-statistics p-value was used to assess the significance of AGF community difference between countries, young versus adult animals, and males versus females, and the sum of squares was used to assess the percentage variance explained by the country of origin for each of the four animal species. Additionally, RDA plots were also constructed using the genera abundance data following center log-ratio transformation (CLR).

Assessing phylosymbiosis patterns. To test for patterns of phylosymbiosis and the presence of a cophylogenetic signal between the animal host and the AGF genera constituting the gut community, we used Procrustes Application to Cophylogenetic Analysis (PACo) through the paco R package. Briefly, the analysis involves the host cophenetic distance matrix (reflecting the phylogenetic relationships between hosts), the AGF cophenetic distance matrix based on the phylogenetic relationship of the different AGF genera to each other, and the AGF genera abundance in the samples. With these three inputs, the analysis then translates the distance matrices of the animal host and the AGF phylogenies into principal coordinates, followed by rotating one set of the coordinates to maximize superimposition on the other. The sum of squared residuals of this superimposition was calculated and used as an indication of congruency between the two sets, with a smaller sum of squared residuals indicating better congruency. A bias-correction step was also added. The analysis produces, besides the bias-corrected sum of squared residuals, a p-value for the goodness of fit between the two phylogenies. Additionally, to assess the sensitivity of PACo analysis to community composition variation among hosts of the same species, we repeated the analysis while subsampling one individual per host genus (n=100 subsamples) and compared the distribution of PACo Procrustes residuals of the sum of squared

differences between different animal species, different animal families, and different gut types. To test for the significance of the difference between residuals, we used Wilcoxon test with Benjamini-Hochberg adjustment.

For pinpointing specific animal host-fungal associations, we employed two approaches. We first used the phyloSignal command in the phylosignal R package to calculate three global phylogenetic signal statistics, Abouheif's Cmean, Moran's I, and Pagel's Lambda. The values of these statistics plus the associated p-values identify the AGF genera that have a significant association with an animal host. We considered any genus with p-value < 0.05 with at least one statistic to be significantly correlated to the host phylogenetic tree. We then used the lipaMoran command in the phylosignal R package to calculate Local Indicator of Phylogenetic Association (LIPA) values for each sample-AGF genus pair, along with the associated p-values of association significance. For AGF genera showing significant associations (LIPA p-values <0.05), we calculated average LIPA values for each animal host species, and animal family. We considered average LIPA values in the range of 0.2-0.4 to represent weak associations, in the range 0.4-1 to represent moderate associations, and above 1 to represent strong associations.

To further explore the notion that enrichments of an ensemble of multiple genera, rather than a single genus, is responsible for the distinct community structure observed in foregut fermenters, we constructed a double principal coordinate analysis (DPCoA) ordination using the genera with abundance in the top 25% in each sample and that occurred in at least 50% of the samples. The genera (n=28) were first selected using the filterfun_sample(topp(0.25)) command in phyloseq followed by pruning the dataset using the genefilter_sample command. The DPCoA was constructed using the ordinate command in phyloseq followed by plot_ordination. DPCoA uses both abundance and phylogenetic information about the samples, allowing both the samples

and the taxa to be plotted on the same coordinate space, and thus the Euclidean distance between samples or their group centroids and AGF genera could be compared. Thus, AGF genera with Euclidean distances close to group centroids are considered to contribute more to the community structure of the group. We used betadisper in the R package vegan to calculate centroids for the three different gut types, the nine different animal families, and the animal genera with at least 4 individuals (n=15), and ggplot2 to draw 95% confidence level ellipses for the three gut types.

Transcriptomic analysis. Prior studies by our research group have generated 21 transcriptomes from 7 genera [27, 28]. Here, we added 20 transcriptomes from 7 additional genera, isolated during a long-term multi-year isolation effort in the authors' laboratory [4, 29] and included an extra 11 publicly available transcriptomic datasets [30, 31, 32, 33]. Cultures grown in rumen fluid-cellobiose medium [34] were vacuum filtered and then grounded with a pestle under liquid nitrogen. Total RNA was extracted using an Epicentre MasterPure yeast RNA purification kit (Epicentre, Madison, WI) according to manufacturer's instructions. Transcriptomic sequencing using Illumina HiSeq2500 platform and 2 × 150 bp paired-end library was conducted using the services of a commercial provider (Novogene Corporation, Beijing, China) or at the Oklahoma State University Genomics and Proteomics Center. The RNA-seq data were quality trimmed and *de novo* assembled with Trinity (v2.6.6) using default parameters. Redundant transcripts were clustered using CD-HIT [35] with identity parameter of 95% (–c 0.95), and subsequently used for peptide and coding sequence prediction using the TransDecoder (v5.0.2) (https://github.com/TransDecoder/TransDecoder) with a minimum peptide length of 100 amino acids. BUSCO [36] was used to assess transcriptome completeness using the fungi_odb10 dataset modified to remove 155 mitochondrial protein families as previously suggested [30]. The dataset of 52 transcriptomes was used for phylogenomic analysis as described in [37]. In addition, five

Chytridiomycota Genomes (*Chytriomyces* sp. strain MP 71, *Entophlyctis helioformis* JEL805, *Gaertneriomyces semiglobifer* Barr 43, *Gonapodya prolifera* JEL478, and *Rhizoclosmatium globosum* JEL800) were included to provide calibration points. The same phylogenomic dataset (670 protein-coding genes) produced for [37] was used as the original input. Gap regions were removed using trimAl v1.4 [38]. Alignment files that contained no missing taxa and were longer than 150 nucleotide sites were selected for subsequent analyses. By employing a greedy search in PartitionFinder v2.1.1 [39], the 88 selected alignments were grouped into 15 partitions with independent substitution models. All partition files and respective models were loaded in BEAUti v1.10.4 [40] with calibration priors specified as previously described [28] ((i) a direct fossil record of Chytridiomycota from the Rhynie Chert (407 Mya) & (ii) the emergence time of Chytridiomycota (573 to 770 Mya as 95% HPD)) for Bayesian inference and divergence time estimation implemented in BEAST v1.10.4. The Birth-Death incomplete sampling tree model was employed for interspecies relationship analyses. Unlinked strict clock models were used for each partition independently. Three independent runs were performed for 50 million generations and Tracer v1.7.1 [41] was used to confirm that sufficient effective sample size (ESS>200) was reached after the default burn-in (10%). The maximum clade credibility (MCC) tree was compiled using TreeAnnotator v1.10.4 [40].

Shotgun metagenomic sequencing. DNA from a subset of samples (n=9) from a cow (n=1), horse (n=1), goats (n=1), sheep (n=1), white-tail deer (n=1), bison (n=1), buffalo (n=1), camel (n=1), and elephant (n=1) was used as a template for metagenomic sequencing on a NextSeq 2K P2 2 × 300-bp paired-end technology with standard library prep using IDT XGen library kit. Sequencing was conducted at the University of Oklahoma Clinical Genomics Facility.

Metagenomic reads processing. FastQC was used to assess the quality of metagenomic reads and Trimmomatic v.0.36 [42] was used to trim reads to remove adapters and remove low-quality reads with a sliding window size of 4 and a sliding window minimum quality of 15. High-quality reads were classified at the phylum level using Kaiju v1.7.3 [43] and a reference database comprised of protein sequences from NCBI nr covering Bacteria, Archaea, Viruses, Fungi, and microbial eukaryotes. Reads were also classified using GOTTCHA2 (https://github.com/poeli/GOTTCHA2) against fungal nucleotide unique signature sequences built from NCBI RefSeq Release 90. High-quality reads were assembled into contigs using IDBA-UD [44] and a minimum contig length of 2000 bp. For binning eukaryotic genomes from the metagenomic assemblies, we used EukRep [45] to identify and output contigs of potential eukaryotic origins. These potential eukaryotic contigs were then binned using CONCOCT v.1.1 [46] with a minimum contig length of 2500 bp, and a Kmer length of 4. The obtained bins were then filtered to remove all bins <1M bp as previously suggested. For all remaining bins, a prokaryotic classifier (GTDB-Tk) [47] was used to identify and classify falsely identified prokaryotic bins. All steps for read processing and assembly (with the exception of EukRep) were run through Kbase [48, 49, 50]. The outline of the pipeline used is shown in Figure S13.

Quantitative PCR. We quantified and compared total AGF to total bacterial load in a subset of the samples comprised of ten cattle, ten goats, ten sheep, and ten horses using quantitative PCR. This was done to further confirm the extremely low relative abundance of AGF DNA when compared to bacterial DNA in the herbivorous feces. The 25-µl PCR reaction volume contained 1 µl of extracted DNA, 0.3 µM of primers AGF-LSU-EnvS primer pair (AGF-LSU-EnvS For: 5'-GCGTTTRRCACCASTGTTGTT-3' and AGF-LSU-EnvS Rev: 5'-GTCAACATCCTAAGYGTAGGTA-3') [7] targeting a ~370 bp region of the LSU rRNA gene

(corresponding to the D2 domain), and SYBR GreenER™ qPCR SuperMix for iCycler™ (ThermoFisher, Waltham, Massachusetts). Reactions were run on a MyiQ thermocycler (Bio-Rad Laboratories, Hercules, CA). The reactions were heated at 95°C for 8.5 min, followed by 40 cycles, with one cycle consisting of 15 sec at 95°C and 1 min at 55°C. Reactions were run in parallel with a pCR 4-TOPO or pCR-XL-2-TOPO plasmid (ThermoFisher, Waltham, Massachusetts) containing an insert spanning ITS1-5.8S rRNA-ITS2-D1/D2 region of 28S rRNA from a pure culture strain as a positive control, as well as to generate a standard curve. The efficiency of the amplification of standards (*E*) was calculated from the slope of the standard curve using the equation $E = (10^{-1/\text{slope}}) - 1$ and was found to be 0.89. Given the variation in the number of rRNA loci in AGF genomes, we opted to quantify AGF in fecal samples as the number of LSU rRNA copies/ g sample, where the number of copies was calculated from the standard curve then converted to copies/g feces based on the volume included in the reaction and the original weight of the fecal samples. Bacterial load was also quantified in the same 40 samples using the 515F and 806R prokaryotic-specific primer pair [51] amplifying 16S rRNA V4 hypervariable region. The number of copies of 16S rRNA in each was calculated from standard curves constructed using pCR 4-TOPO plasmid (ThermoFisher, Waltham, Massachusetts) containing an insert spanning the full 16S rRNA from a pure culture strain, then converted to copies/g feces based on the volume included in the reaction and the original weight of the fecal samples.

II. Supplementary figures.

Figure S1: Rarefaction curve Rarefaction curves showing the increase in the number of genera observed as the number of sequences increases per sample. Rarefaction data were calculated and plotted in R using rarecurve in vegan.

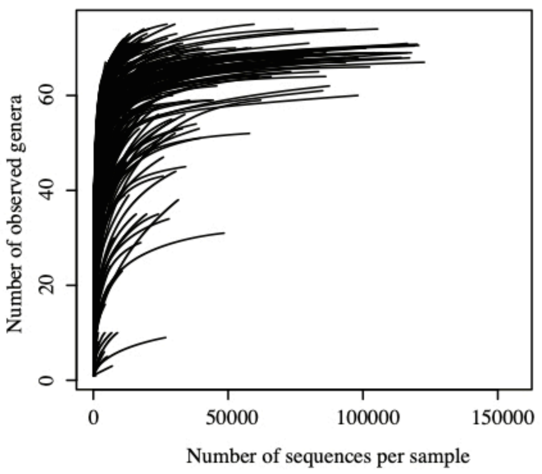

Number of observed genera
0
20
40
60
0
50000
100000
150000
Number of sequences per sample

Figure S2: Occurrence and relative abundance distribution of all AGF genera encountered in this study. (A) Number of samples (out of 661) in which each AGF genus was identified. Genera are shown in descending order of their average percentage abundance across samples. (B) Box and whisker plot showing the distribution of the percentage abundance of the 84 genera. Genera are shown in the same order as in (A).

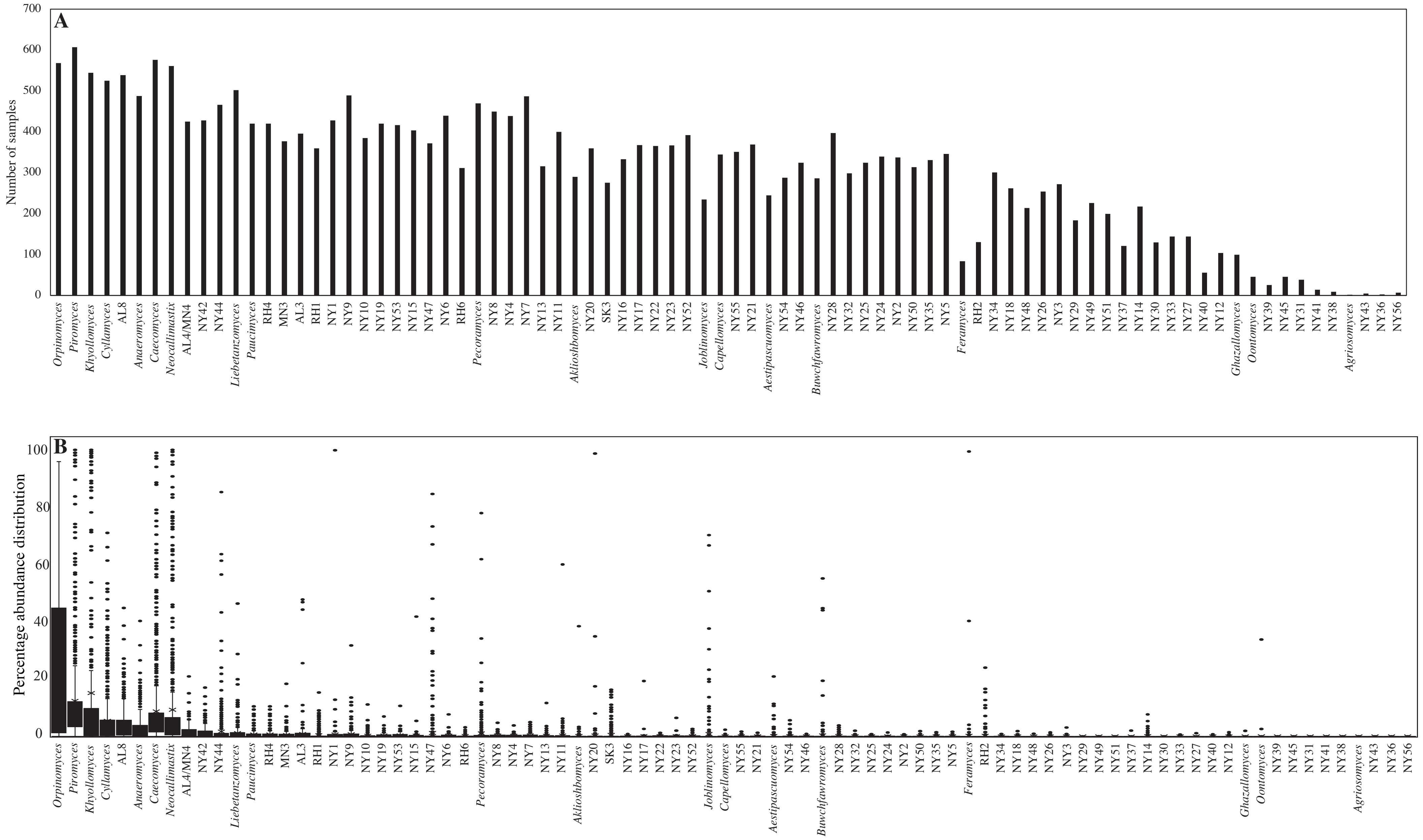
A
Number of samples
B
Percentage abundance distribution
Orpinomyces
Piromyces
Khyollomyces
Cyllamyces
AL8
Anaeromyces
Caecomyces
Neocallimastix
AL4/MN4
NY42
NY44
Liebetanzomyces
Paucimyces
RH4
MN3
AL3
RH1
NY1
NY9
NY10
NY19
NY53
NY15
NY47
NY6
RH6
Pecoramyces
NY8
NY4
NY7
NY13
NY11
Aklioshbomyces
NY20
SK3
NY16
NY17
NY22
NY23
NY52
Joblinomyces
Capellomyces
NY55
NY21
Aestipascuomyces
NY54
NY46
Buwchfawromyces
NY28
NY32
NY25
NY24
NY2
NY50
NY35
NY5
Feramyces
RH2
NY34
NY18
NY48
NY26
NY3
NY29
NY49
NY51
NY37
NY14
NY30
NY33
NY27
NY40
NY12
Ghazallomyces
Oontomyces
NY39
NY45
NY31
NY41
NY38
Agriosomyces
NY43
NY36
NY56

Figure S3: Abundance-occurrence plots. Relationship between occurrence (number of samples) and average relative abundance of each of the 84 genera encountered in this study. The number of samples in which the genera were identified is shown on the X-axis. Average percentage abundance across samples is plotted on the Y axis in a logarithmic scale to show genera present below 1% abundance. Occurrence and relative abundance of different genera were largely correlated (R^2=0.71), with the few highlighted exceptions (*Joblinomyces*, *Feramyces*, *Onotomyces*, and RH2).

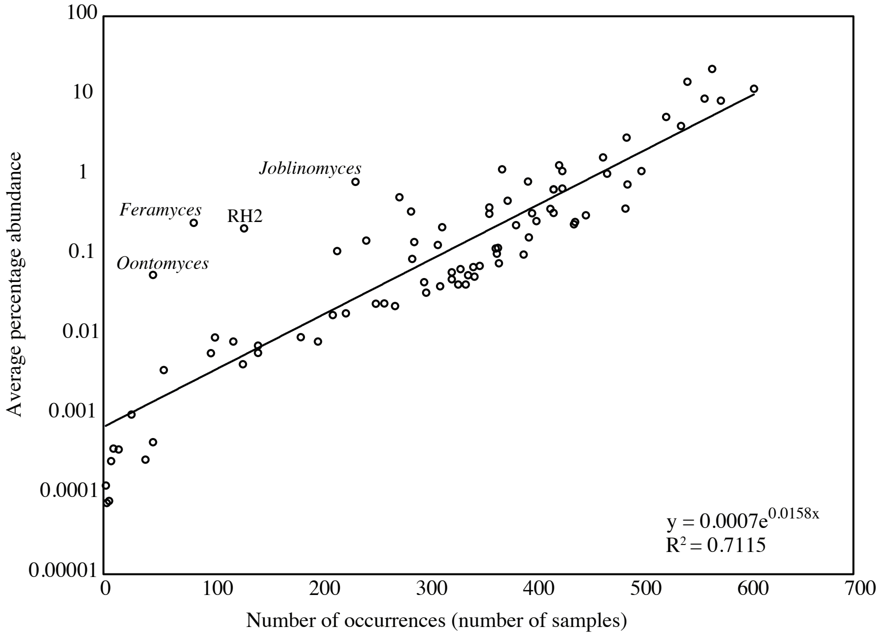

Average percentage abundance
100
10
1
0.1
0.01
0.001
0.0001
0.00001
0
100
200
300
400
500
600
700
Number of occurrences (number of samples)
Joblinomyces
Feramyces
RH2
Oontomyces
y = 0.0007e^0.0158x
R² = 0.7115

Figure S4: Comparison of community structure patterns between Illumina- and SMRT PacBio-generated datasets. Comparative analysis of the community structure and composition in 61 samples (60 cows and 1 bison) that were amplified using the D2-targeting primers and sequenced with Illumina sequencing technology, as well as using a different set of primers targeting the whole D1/D2 region and sequenced using the PacBio sequencing technology.
(A) AGF community composition in the 61 samples sequenced using Illumina (left stacked bar) and SMRT (right stacked bar) sequencing technologies showing the overall similarity in community composition. (B-D) Community structure in the 61 samples sequenced with the two sequencing technologies. (B) Canonical correspondence analysis (CCA) of the AGF community in the 122 samples (61 samples x 2 sequencing technologies) showing the similarity in community between Illumina-sequenced (red) and SMRT-sequenced (blue) samples. The community structures of the same sample sequenced with the two sequencing technologies were similar (clustered close to each other on the CCA plot). This is evident from the comparisons of the distribution of Euclidean distances on the CCA plot between all possible pairs of Illumina-sequenced samples (red), all possible pairs of SMRT-sequenced samples (blue), and the 61 pairs of Illumina versus PacBio sequenced samples (grey) (C), where the Euclidean distances between the Illumina-SMRT pairs are smaller. Boxplots extend from the first to the third quartile and the median is shown as a thick line in the middle. The whiskers extending on both ends represent variability outside the quartiles and are calculated as follows: Minimum whisker = minimum quartile - 1.5 x inter-quartile range; Maximum whisker = maximum quartile + 1.5 x inter-quartile range. All points outside the box and whiskers are outliers. A total of 1830 data points were used to calculate the first two box and whisker plots (all possible pairs of Illumina-sequenced samples, and all possible pairs of SMRT-sequenced samples), while the last box and whisker plot was constructed using 61 data points corresponding to all 61 Illumina-SMRT pairs. Data for the grey

box and whisker plot in (C) (the 61 Illumina-SMRT pairs) is shown in detail in (D). Fifty-two pairs lie within a Euclidean distance of 1 from each other, and only 9 pairs lie within a higher Euclidean distance from each other, attesting to the similarity in community composition between the pairs originating from the same sample.

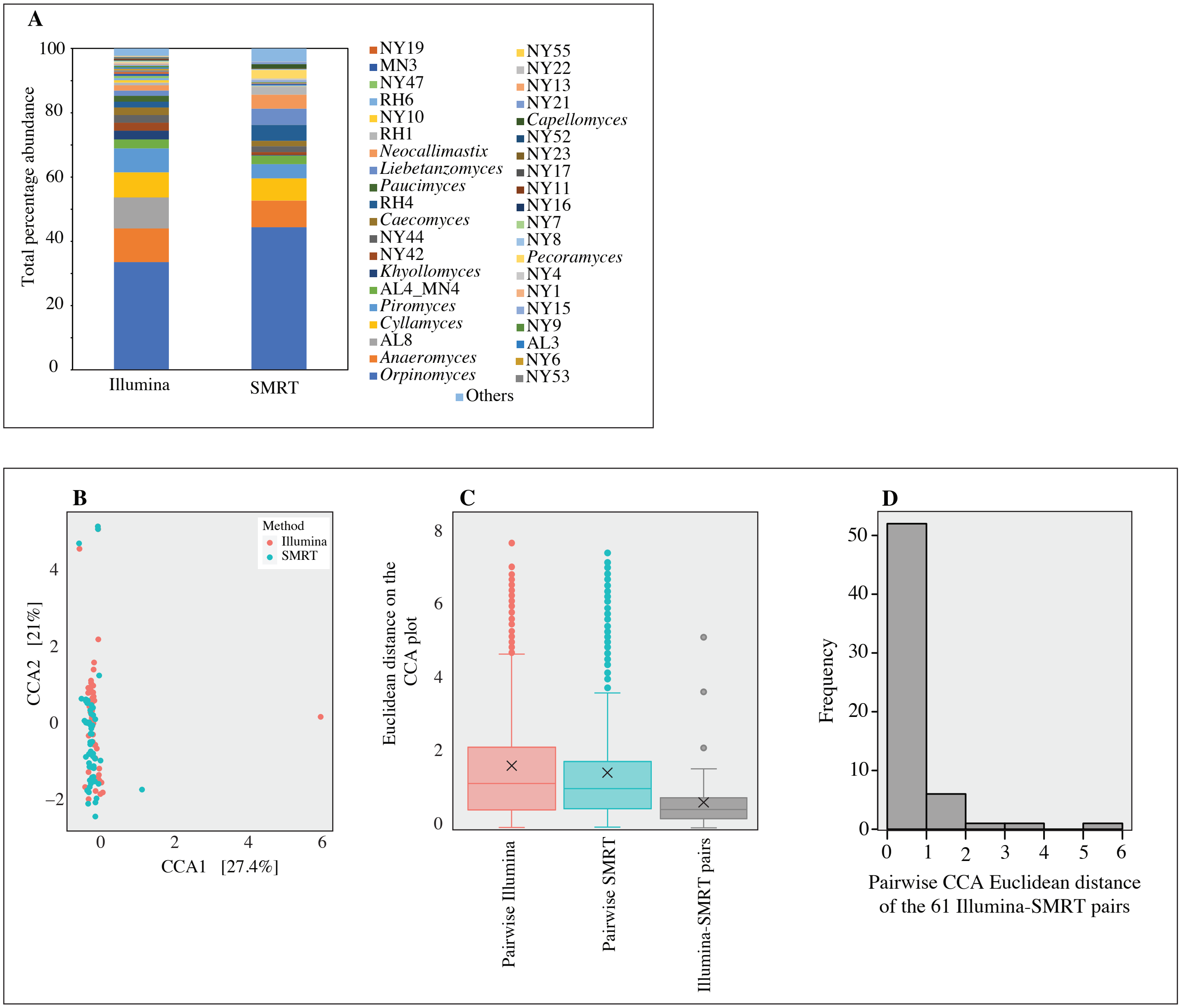
A
Total percentage abundance
100
80
60
40
20
0
Illumina
SMRT
NY19
MN3
NY47
RH6
NY10
RH1
Neocallimastix
Liebetanzomyces
Paucimyces
RH4
Caecomyces
NY44
NY42
Khyollomyces
AL4_MN4
Piromyces
Cyllamyces
AL8
Anaeromyces
Orpinomyces
NY55
NY22
NY13
NY21
Capellomyces
NY52
NY23
NY17
NY11
NY16
NY7
NY8
Pecoramyces
NY4
NY1
NY15
NY9
AL3
NY6
NY53
Others
B
Method
Illumina
SMRT
CCA2 [21%]
CCA1 [27.4%]
C
Euclidean distance on the CCA plot
Pairwise Illumina
Pairwise SMRT
Illumina-SMRT pairs
D
Frequency
Pairwise CCA Euclidean distance of the 61 Illumina-SMRT pairs

Figure S5. Confirmation of the unique position of novel AGF genera (identified using D2 LSU amplicons and Illumina sequencing) using longer D1/D2 LSU amplicons and PacBio sequencing. Maximum likelihood phylogenetic tree constructed using the alignment of the D1/D2 region from representatives of all cultured (blue) and uncultured (orange) genera, the D1/D2 region from representatives of the 49 novel genera (green) identified by PacBio sequencing in this study and the D2 region from representatives of the 7 novel genera that were not identified in the PacBio dataset. Clades of genera are color-coded by family as shown in the labels around the tree (for the newly proposed families *Neocallimastigaceae*, *Caecomycetaceae*, *Piromycetaceae*, and *Anaeromycetaceae*). Putative novel families encompassing multiple of the novel genera identified here, as well as genera previously unaffiliated with the above four families are shown in red labels around the tree. These include novel families affiliated with the genus *Khoyollomyces*, the genus *Joblinomyces*, the genera *Buwchfawromyces* and *Tahromyces*, the genus *Aklioshbomyces*, and the genus *Paucimyces*. Bootstrap support is shown as black dots for nodes with >70% support.

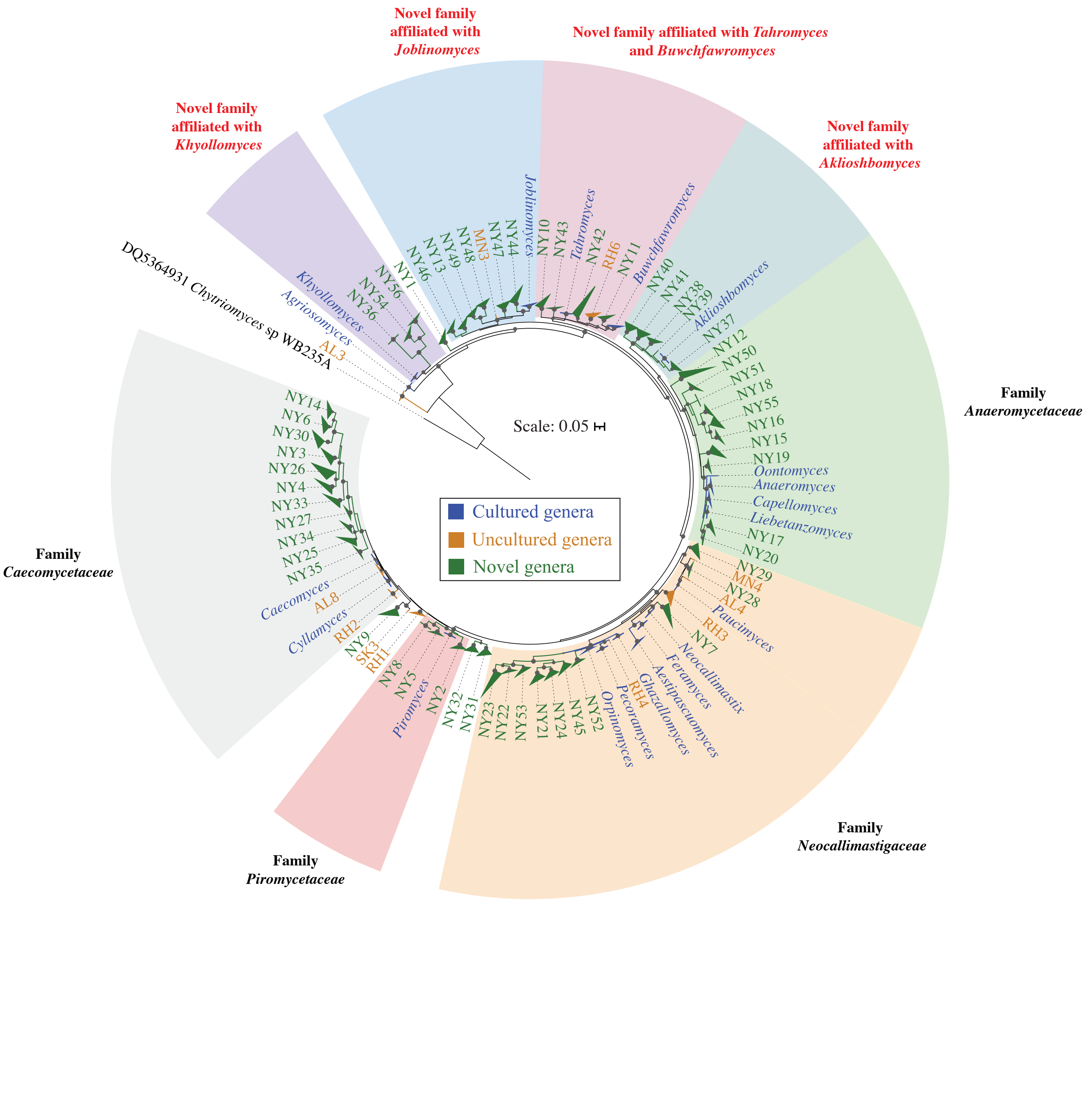

Novel family affiliated with *Joblinomyces*
Novel family affiliated with *Tahromyces* and *Buwchfawromyces*
Novel family affiliated with *Khyollomyces*
Novel family affiliated with *Aklioshbomyces*
Family *Anaeromycetaceae*
Family *Caecomycetaceae*
Family *Neocallimastigaceae*
Family *Piromycetaceae*
DQ5364931 Chytriomyces sp WB235A
Scale: 0.05
Cultured genera
Uncultured genera
Novel genera

Figure S6. Comparison of the topologies of the two maximum likelihood phylogenetic trees constructed using the alignment of the D2 region obtained via Illumina sequencing (left) versus the D1/D2 region obtained using PacBio sequencing (right). Both trees show representatives of all cultured, uncultured, and the 49 novel genera that were identified in both the Illumina and the PacBio datasets. The 7 novel genera that were not identified in the PacBio dataset are shown in red text (only in the Illumina tree). Families and putative families are color coded using the same color code in Figure 2a and Figure S5. The majority of genera (79 out of 87 total) had a similar topology in both trees (i.e., belonged to the same family or putative family). The 8 genera with discrepant topology in the two trees are shown in boldface. The trees were rooted using *Chytriomyces* sp. WB235A as an outgroup (not shown).

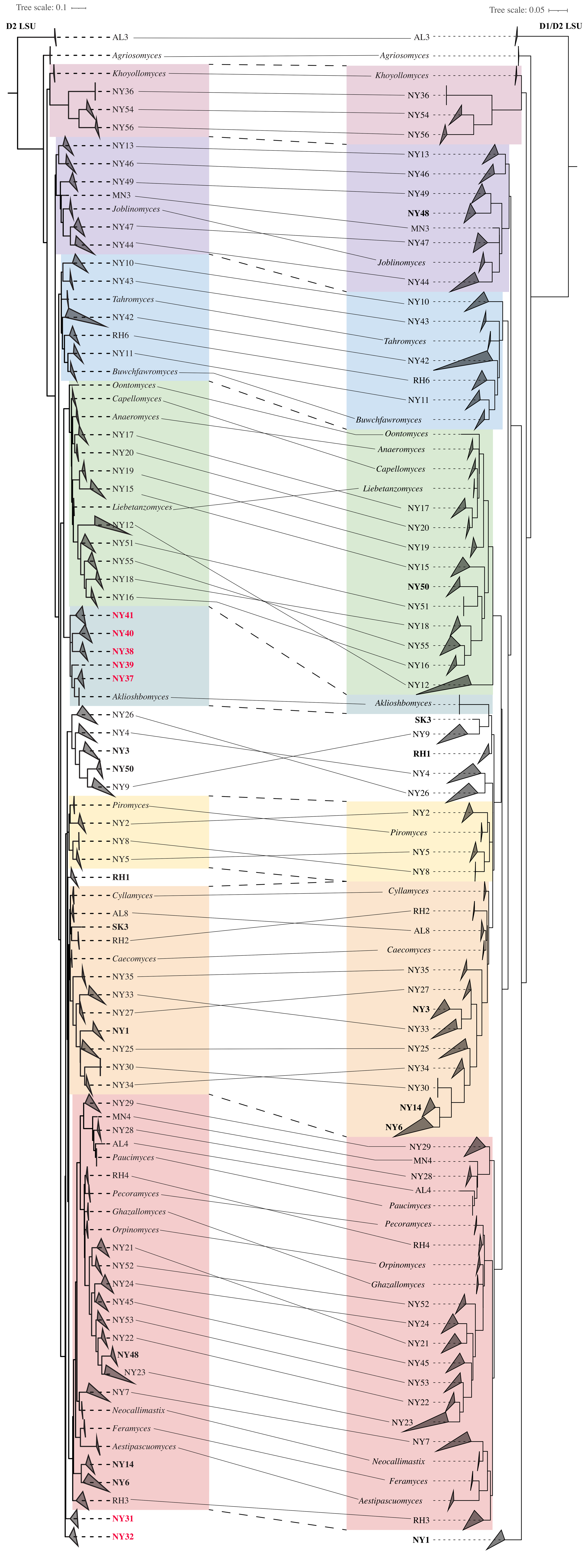

Tree scale: 0.1
D2 LSU
Tree scale: 0.05
D1/D2 LSU
AL3
Agriosomyces
Khoyollomyces
NY36
NY54
NY56
NY13
NY46
NY49
MN3
Joblinomyces
NY47
NY44
NY10
NY43
Tahromyces
NY42
RH6
NY11
Buwchfawromyces
Oontomyces
Capellomyces
Anaeromyces
NY17
NY20
NY19
NY15
Liebetanzomyces
NY12
NY51
NY55
NY18
NY16
NY41
NY40
NY38
NY39
NY37
Aklioshbomyces
NY26
NY4
NY3
NY50
NY9
Piromyces
NY2
NY8
NY5
RH1
Cyllamyces
AL8
SK3
RH2
Caecomyces
NY35
NY33
NY27
NY1
NY25
NY30
NY34
NY29
MN4
NY28
AL4
Paucimyces
RH4
Pecoramyces
Ghazallomyces
Orpinomyces
NY21
NY52
NY24
NY45
NY53
NY22
NY48
NY23
NY7
Neocallimastix
Feramyces
Aestipascuomyces
NY14
NY6
RH3
NY31
NY32
AL3
Agriosomyces
Khoyollomyces
NY36
NY54
NY56
NY13
NY46
NY49
NY48
MN3
NY47
Joblinomyces
NY44
NY10
NY43
Tahromyces
NY42
RH6
NY11
Buwchfawromyces
Oontomyces
Anaeromyces
Capellomyces
Liebetanzomyces
NY17
NY20
NY19
NY15
NY50
NY51
NY18
NY55
NY16
NY12
Aklioshbomyces
SK3
NY9
RH1
NY4
NY26
NY2
Piromyces
NY5
NY8
Cyllamyces
RH2
AL8
Caecomyces
NY35
NY27
NY3
NY33
NY25
NY34
NY30
NY14
NY6
NY29
MN4
NY28
AL4
Paucimyces
Pecoramyces
RH4
Orpinomyces
Ghazallomyces
NY52
NY24
NY21
NY45
NY53
NY22
NY23
NY7
Neocallimastix
Feramyces
Aestipascuomyces
RH3
NY1

Fig. S7. Patterns of AGF alpha diversity. Samples with at least 1000 sequences were included (n=421); and the analysis was repeated with and without randomly subsampling 1000 sequences from each sample. Alpha diversity patterns were assessed using four different indices (observed number of genera, Shannon, Simpson, and Inverse Simpson) and the two sampling strategies. (A) Box and whisker plots showing the distribution of 4 alpha diversity measures for different animal species (top row), animal families (middle row), and animal gut types (bottom row). Boxplots extend from the first to the third quartile and the median is shown as a thick line in the middle. The whiskers extending on both ends represent variability outside the quartiles and are calculated as follows: Minimum whisker = minimum quartile - 1.5 x inter-quartile range; Maximum whisker = maximum quartile + 1.5 x inter-quartile range. All points outside the box and whiskers are outliers. The number of data points used to calculate each box and whisker plot correspond to the number of samples belonging to each animal species, and animal family as defined in Figure 1D, or the number of samples belonging to each gut type as shown in Figure 2D. (B) Results of two-tailed ANOVA showing the significant effect of the host species, animal family, and animal gut type, but not domestication status, on alpha diversity measures regardless of the index used or the subsampling approach ($p<0.0002$). (C) Tukey test results for two-tailed pairwise animal species, animal family, and animal gut type comparisons (8 comparisons each; 2 subsampling approaches x 4 diversity indices). Specifically, hindgut animals harbored a significantly less diverse community compared to foregut ruminants (in all 8 comparisons; p-value <0.00001). Accordingly, members of the hindgut family Equidae harbored a less diverse community when compared to the foregut ruminant families Cervidae and Bovidae (in 6/8 comparisons; p-value <0.002, and in 6/8 comparisons; p-value <0.00004, respectively); and horse communities were significantly less diverse than these of deer (in 6/8 comparisons; p-value <0.04), cattle (in 6/8 comparisons; p-value <0.00004), goats (in 6/8 comparisons; p-value <0.02), and sheep (in 4/8 comparisons; p-value <0.01). Within foregut ruminants, communities in animals

belonging to the families Cervidae and Bovidae were not significantly different (7/8 comparisons; p-value >0.09). As well, on the animal host species level, most comparisons indicated no significant differences in diversity between deer, goat, cattle, and sheep.

A

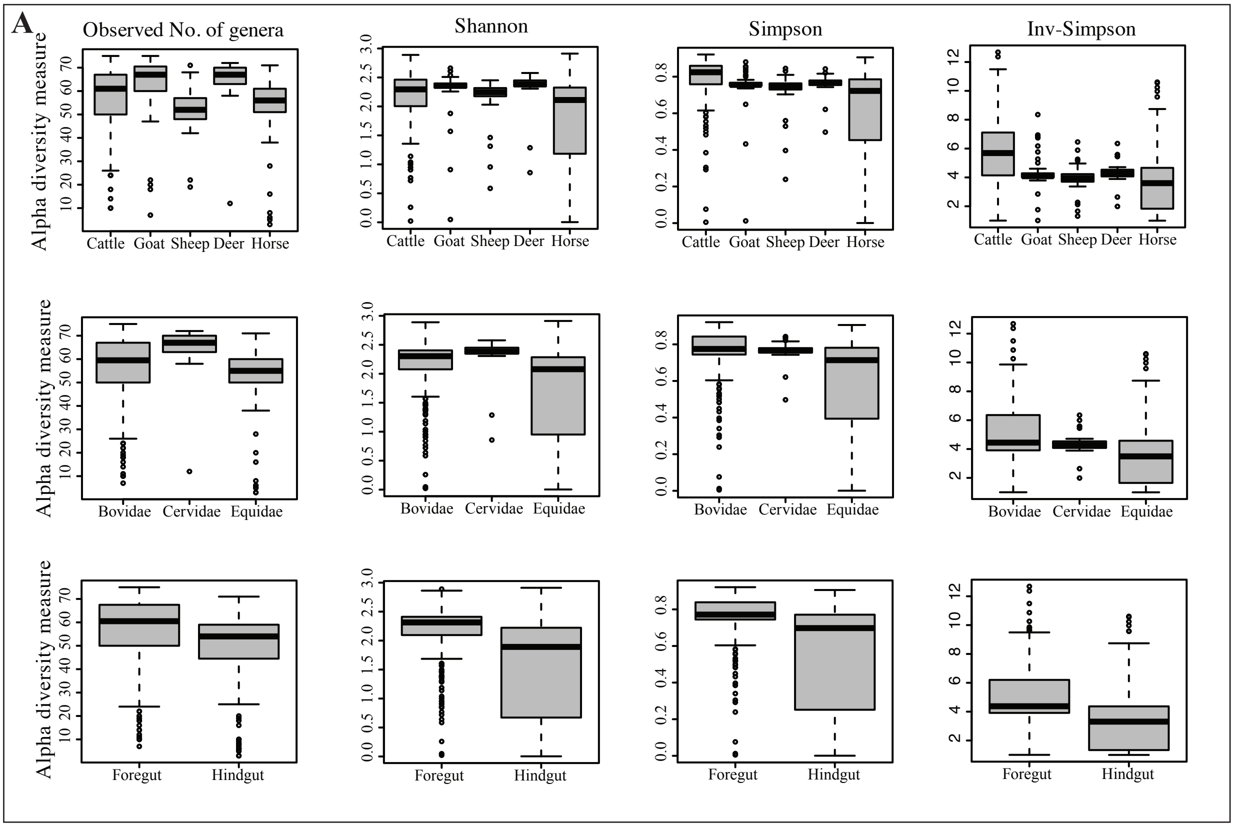

B

Host factor	Observed No. of genera		Shannon		Simpson		Inv-Simpson	
	With subsampling	Without subsampling	With subsampling	Without subsampling	With subsampling	Without subsampling	With subsampling	Without subsampling
Animal species	1.7E-15	1.4E-06	8.4E-06	0.0001	8E-05	0.0001	9.9E-08	4.2E-08
Family	2E-16	2E-16	2.2E-15	1E-14	1.5E-14	4.8E-13	1.6E-06	9E-07
Gut type	6.5E-10	9.2E-12	2E-16	2E-16	2E-16	2E-16	1.5E-08	3.2E-08
Domestication	NS	NS	NS	NS	NS	NS	NS	NS

C

Host factor	Group1	Group 2	With subsampling				Without subsampling			
			Observed No. of genera	Shannon	Simpson	Inv-Simpson	Observed No. of genera	Shannon	Simpson	Inv-Simpson
Animal Species	Deer	Cattle	0.0000002	0.99	1	0.3	0.43	0.99	1	0.3
	Goat	Cattle	0	0.95	1	0.051	0.34	0.94	1	0.049
	Goat	Deer	1	1	1	1	1	1	1	1
	Horse	Cattle	0.99	0.00004	0	0.0000003	0.97	0.00004	0	0.0000002
	Horse	Deer	0.0003	0.0009	0.015	0.99	0.04	0.0008	0.015	0.99
	Horse	Goat	0.000007	0.000004	0.0001	0.99	0.015	0.000004	0.0001	0.99
	Sheep	Cattle	0.00004	1	0.97	0.00002	0.92	1	0.96	0.00002
	Sheep	Deer	0.89	0.99	1	0.99	0.03	0.99	1	0.99
	Sheep	Goat	0.9	0.99	0.99	0.99	0.013	0.99	0.99	0.99
	Sheep	Horse	0.079	0.003	0.01	1	1	0.005	0.001	1
Animal Family	Cervidae	Bovidae	0.0006	0.84	1	0.86	0.09	0.83	1	0.86
	Equidae	Bovidae	0.47	0	0	0.00004	0.11	0	0	0.00003
	Equidae	Cervidae	0.00002	0.000008	0.0001	0.91	0.001	0.000006	0.0001	0.90
Gut type	Hindgut	Foregut	0.00001	0	0	0.0000002	0.0000006	0	0	0.0000002

Figure S8. Ordination plots based on AGF community structure in the 661 samples studied here. (A) RDA plot constructed using the center log-ratio transformed genera abundance data. The % variance explained by the first two axes are displayed on the axes. (B) Non-metric dimensional scaling (NMDS) plots based on the phylogenetic similarity-based (unweighted and weighted Unifrac) indices as shown above each plot. NMDS stress value is shown in the upper corner of each plot. (C) Principal coordinate analysis (PCoA) plot based on the phylogenetic similarity-based index unweighted Unifrac. The % variance explained by the first two axes are displayed on the axes. Samples are color-coded by animal species, while the shape depicts the gut type.

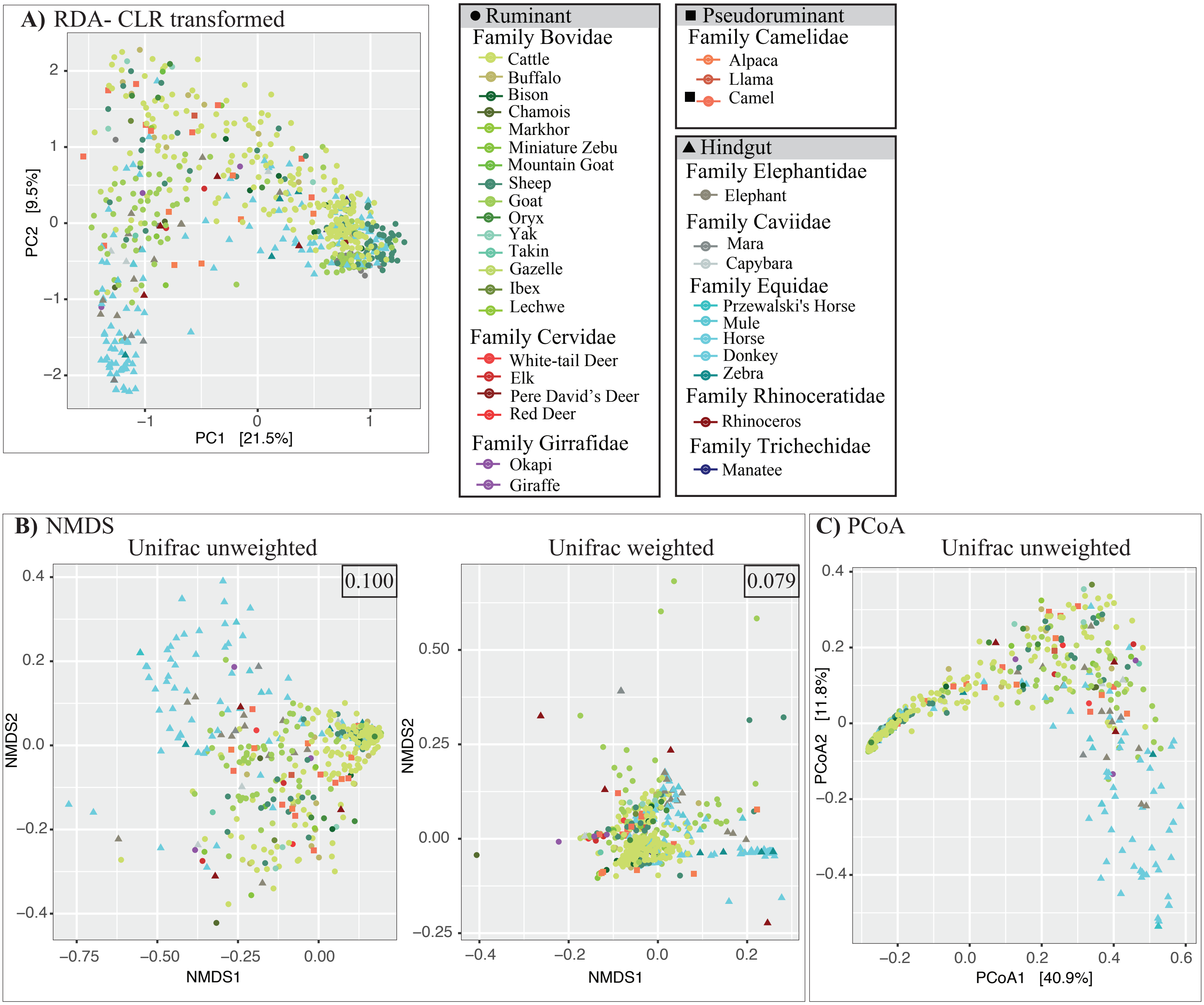

A) RDA- CLR transformed
PC1 [21.5%]
PC2 [9.5%]
● Ruminant
Family Bovidae
Cattle
Buffalo
Bison
Chamois
Markhor
Miniature Zebu
Mountain Goat
Sheep
Goat
Oryx
Yak
Takin
Gazelle
Ibex
Lechwe
Family Cervidae
White-tail Deer
Elk
Pere David's Deer
Red Deer
Family Girrafidae
Okapi
Giraffe
■ Pseudoruminant
Family Camelidae
Alpaca
Llama
Camel
▲ Hindgut
Family Elephantidae
Elephant
Family Caviidae
Mara
Capybara
Family Equidae
Przewalski's Horse
Mule
Horse
Donkey
Zebra
Family Rhinoceratidae
Rhinoceros
Family Trichechidae
Manatee
B) NMDS
Unifrac unweighted
0.100
Unifrac weighted
0.079
NMDS1
NMDS2
C) PCoA
Unifrac unweighted
PCoA1 [40.9%]
PCoA2 [11.8%]

Figure S9. (A) Box and whisker plots of the distribution of PCoA ordination distance of each sample to its group centroid (pink) and all other centroids (non-group, cyan). The PCoA plots are shown in Fig. 4a. Centroids were calculated using the command betadisper in the vegan package for each animal species (left, only for animals with 4 or more individuals), animal family (middle), and animal gut type (right), while ordination distance between each point and the group centroid was calculated as the Euclidean distance between two points in an ordination plot. Boxplots extend from the first to the third quartile and the median is shown as a thick line in the middle. The whiskers extending on both ends represent variability outside the quartiles and are calculated as follows: Minimum whisker = minimum quartile - 1.5 x inter-quartile range; Maximum whisker = maximum quartile + 1.5 x inter-quartile range. All points outside the box and whiskers are outliers. The number of data points used to calculate each box and whisker plot to a group centroid corresponds to the number of samples belonging to each animal species, animal family, or animal gut type as defined in Figure 1D and 2D. The number of data points used to calculate the box and whisker plot to a non-group centroid differed and is equal to the number of samples in each group multiplied by the total number of groups minus 1 (for example, the number of data points to the non-alpaca group centroid is 90: 6 (total number of alpacas) x [16 (total number of animal species compared) -1]. In general, samples clustered by the animal species (small variation in Euclidean distance to the animal species centroid), with only a few animals (e.g. horse, donkey, sheep, goat) showing large variation from their respective animal species centroid, usually due to only a few divergent samples. Interestingly, the microbial community structure showed a higher level of "variability" in hindgut animals when compared to foregut. Samples from animals belonging to the foregut families Bovidae, Cervidae, Giraffidae, and Camelidae clustered close to their respective animal family centroid. However, large variation was observed for the hindgut families Equidae, and Caviidae. This was also

observed when comparing samples to their respective gut type centroid, where samples from foregut animals clustered close to their gut type centroid, while samples from hindgut animals showed large variation in their distance from the hindgut centroid. (B) Quantitative assessment of host factors affecting community structure via two-tailed multivariate regression methods (multiple regression of matrices (MRM), Mantel tests for matrices correlations, and Procrustes rotation). Methods compared the AGF community dissimilarity matrix (Unifrac weighted (W), Unifrac unweighted (UW), Bray-Curtis, and Jaccard), to a matrix of each of the host factors tested (animal species, animal family, animal gut type, and domestication status). For the MRM analysis, results of the whole model (without partitioning variances into different host factors) are shown on top. The model was found to be significant regardless of the index used. For each of the three methods, the significance of correlation (depicted as the test p-value), and the degree the host factor is affecting community structure (depicted by the R2 regression coefficients of the MRM analysis, the Spearman correlation coefficients of the Mantel test, and the symmetric orthogonal Procrustes statistic of the Procrustes analysis) are shown. Significant p-values (<0.05) are shown in red text. Results of matrices correlation (12 total correlations; 3 methods x 4 dissimilarity indices) using each of the three methods, and regardless of the index used, confirmed the importance of animal host species, family, and gut type in explaining the AGF community structure. Animal host species and family were both found to be significant in all 12 correlations (p-value=0.001 for animal species, and <0.025 for animal family), while the animal gut type was found to be significant in 10 out of the 12 correlations (p-value <0.025). Further, comparing the correlation coefficients produced by each of the methods showed that the animal species explains more of the community structure (as evident by the higher R2 regression coefficients of the MRM analysis, the higher Spearman correlation coefficients of the Mantel test, and the higher symmetric orthogonal Procrustes statistic of the Procrustes

analysis) than the animal family or the gut type. This was true for 10 out of the 12 correlations. On the other hand, domestication status was only found significant in 3 out of the 12 total correlations, albeit with very low correlation coefficients.

A

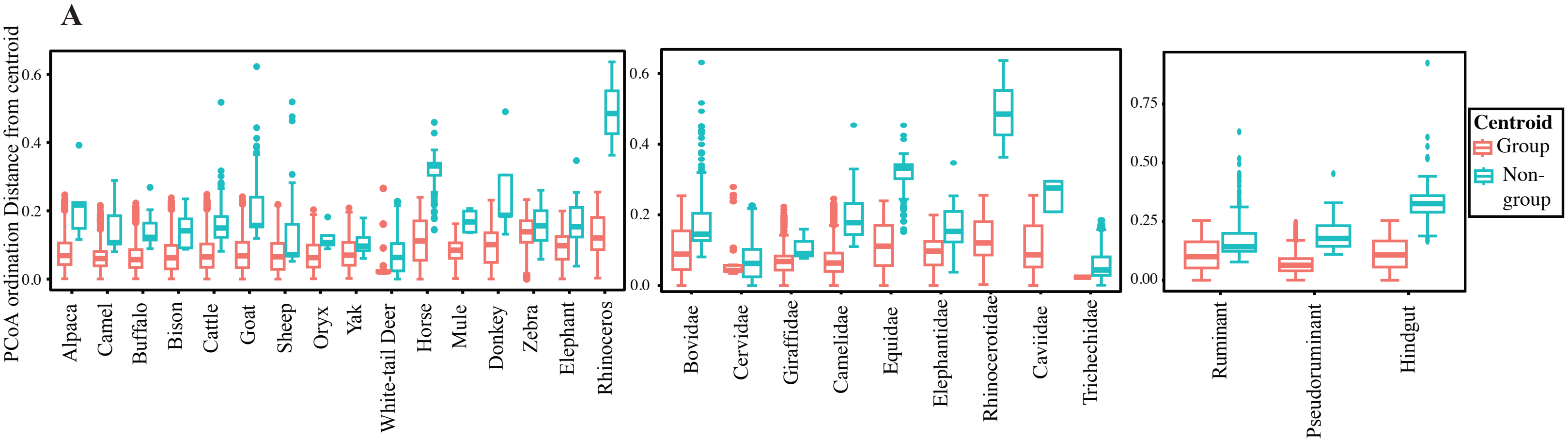

B

Test	Factors	Statistic	Beta diversity index			
			Unifrac (W)	Unifrac (UW)	Bray-Curtis	Jaccard
MRM	Whole model	R^2	0.147	0.050	0.041	0.039
		p-value	**0.001**	**0.001**	**0.001**	**0.001**
		F	9431.8	2869.4	2315.1	2206.3
		F.p-value	**0.001**	**0.001**	**0.001**	**0.001**
	Animal species	p-value	**0.001**	**0.001**	**0.001**	**0.001**
	Host family		**0.024**	**0.015**	**0.001**	**0.001**
	Gut Type		**0.001**	**0.023**	0.993	0.952
	Domestication		0.845	0.804	0.792	0.629
	Animal species	Coefficients	0.342	0.178	0.199	0.252
	Host family		0.306	0.160	0.273	0.159
	Gut Type		0.120	0.052	0.000	-0.001
	Domestication		-0.001	0.004	0.002	0.003
Mantel	Animal species	p-value	**0.001**	**0.001**	**0.001**	**0.001**
	Host family		**0.001**	**0.001**	**0.001**	**0.001**
	Gut Type		**0.001**	**0.001**	**0.001**	**0.001**
	Domestication		**0.036**	0.868	0.285	0.246
	Animal species	Coefficients	0.377	0.217	0.182	0.175
	Host family		0.329	0.173	0.120	0.113
	Gut Type		0.371	0.208	0.156	0.148
	Domestication		0.051	0.027	0.019	0.020
Procrustes	Animal species	p-value	**0.001**	**0.001**	**0.001**	**0.001**
	Host family		**0.001**	**0.001**	**0.001**	**0.001**
	Gut Type		**0.001**	**0.001**	**0.001**	**0.001**
	Domestication		0.082	0.216	**0.033**	**0.013**
	Animal species	Correlation in a symmetric Procrustes	0.507	0.305	0.279	0.272
	Host family		0.448	0.252	0.208	0.203
	Gut Type		0.491	0.491	0.219	0.210
	Domestication		0.061	0.048	0.065	0.075
	Animal species	Procrustes Sum of Squares	0.743	0.743	0.922	0.926
	Host family		0.799	0.937	0.957	0.959
	Gut Type		0.759	0.911	0.952	0.956
	Domestication		0.996	0.998	0.996	0.994

Figure S10: Fungal genera-animal host preferences. Double principal coordinate analysis (DPCoA) biplot constructed using the genera with abundance in the top 25% of each sample and that were encountered in at least 50% of the samples (n=28 genera). DPCoA uses both abundance and phylogenetic information about the samples, allowing both the samples and the taxa to be plotted on the same coordinate space, and thus the ordination distance between samples or their centroids and AGF genera could be compared. AGF genera with ordination distances close to sample centroids are abundant in these samples, and, therefore, contribute more to the community structure. For ease of visualization, individual samples are removed and only the animal species (hexagons, only for animals with 4 or more individuals), animal family (squares), and animal gut type (X) centroids are plotted, with standard deviation data ellipse shown for only the gut type. Centroids and data ellipses were generated using the command betadisper in the vegan package. Animals are color-coded by their respective family as shown in the key and colors follow the same scheme as in Fig 1d. The AGF genera are shown as purple circles. The first two axes explained 69.4% of the variance. There was a clear separation of the hindgut families Equidae (orange square centroid), Rhinocerotidae (green square centroid), from the foregut families Bovidae (pink square centroid), Cervidae (yellow square centroid), and Giraffidae (red square centroid), with the pseudoruminant family Camelidae (purple square centroid) occupying an intermediate position. Of the 28 most abundant genera, 14 fell within the foregut ruminant ellipses, all of which were shared with the overlapping foregut pseudoruminant ellipse. Three additional genera fell within the foregut pseudoruminant ellipse. Only 9 genera fell within the hindgut ellipse outside the foregut ruminant and pseudoruminant ellipses. These genera included the Equidae-specific *Khoyollomyces* and AL3, the Trichechidae-specific *Paucimyces*, and the Rhinocerotidae-specific NY15. The genera *Orpinomyces*, and NY53 fell

outside all ellipses, consistent with their high LIPA values of association with almost all families, hence their intermediary position on the biplot. Several genera, NY42, NY6, NY7, AL4/MN4, *Cyllamyces*, and AL8, fell within the ellipses of all gut types. These genera had moderate LIPA association values with almost all animal genera. The two genera *Piromyces*, and *Caecomyces* also fell within the ellipses of all gut types, but their position is most probably due to their strong association with the family Elephantidae.

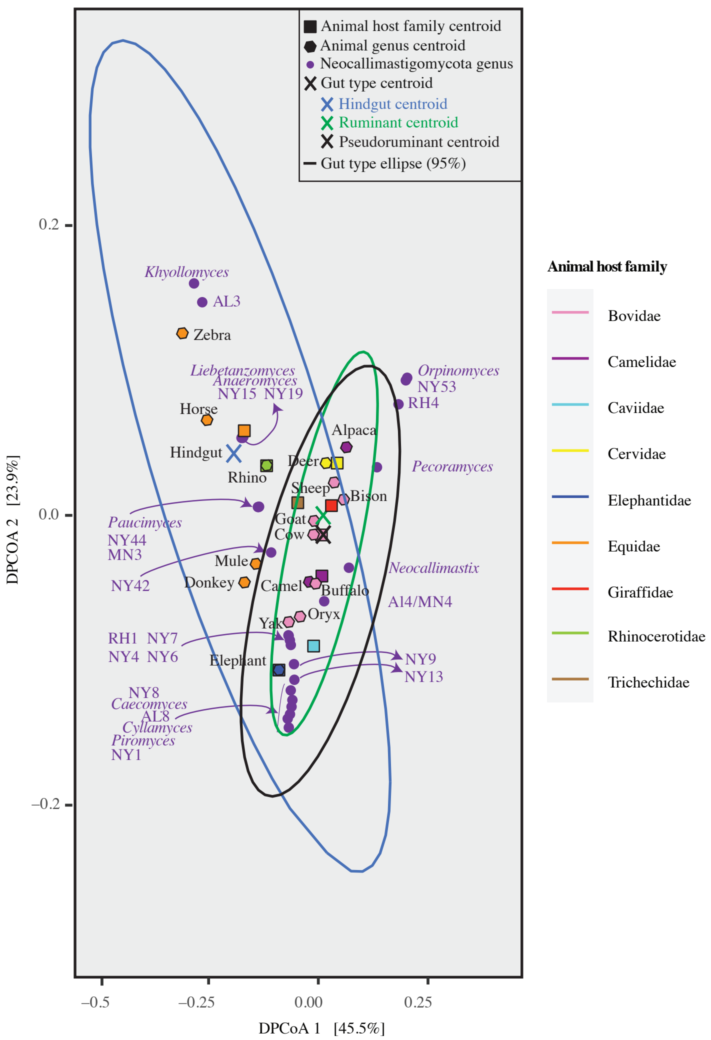

Animal host family centroid
Animal genus centroid
Neocallimastigomycota genus
Gut type centroid
Hindgut centroid
Ruminant centroid
Pseudoruminant centroid
Gut type ellipse (95%)
Khyollomyces
AL3
Zebra
Liebetanzomyces
Anaeromyces
NY15 NY19
Orpinomyces
NY53
RH4
Horse
Alpaca
Hindgut
Deer
Pecoramyces
Rhino
Sheep
Bison
Goat
Cow
Paucimyces
NY44
MN3
NY42
Mule
Donkey
Camel
Buffalo
Neocallimastix
Al4/MN4
Oryx
Yak
RH1 NY7
NY4 NY6
Elephant
NY9
NY13
NY8
Caecomyces
AL8
Cyllamyces
Piromyces
NY1
0.2
0.0
−0.2
−0.5
−0.25
0.00
0.25
DPCOA 2 [23.9%]
DPCoA 1 [45.5%]
Animal host family
Bovidae
Camelidae
Caviidae
Cervidae
Elephantidae
Equidae
Giraffidae
Rhinocerotidae
Trichechidae

Figure S11: Effect of biogeography, age, and sex on AGF community structure in cattle (A, E, I), goats (B, F, J), sheep (C, G, K), and horses (D, H, L). For testing the effect of biogeography, age, and sex on community structure, we opted to carry out comparisons only on samples belonging to the same animal species in an attempt to control for other host-associated factors that might conflate the results. For these comparisons, only the four most-sampled animal species (cattle, goats, sheep, and horses) were considered. RDA ordination plots constructed using the center log-ratio transformed genera abundance data are shown. The first two PCoA axes are plotted, and the percentage variance explained by each axis is shown for each plot. Samples are color-coded (as shown in the figure color key) by their country of origin (A-D), age (E-H), and sex (I-L). For biogeography effects, samples originated from different geographical locations (Argentina, Austria, Czech Republic, Egypt, Germany, Italy, Nepal, New Zealand, and the USA). For age, animals were classified as young (<1 year), or adult (>1 year), and sex was either male or female. Samples where this information was not available are shown as NA. The first two axes explained 46.1%-55.5% of the variance depending on the animal subset. (M) To test for the significance of the above three factors in describing AGF community structure in each animal genus, two-tailed PERMANOVA tests were run using the vegan command adonis. The F- statistics p-value was used to assess the significance of AGF community difference between countries, young versus adult animals, and males versus females, and the sum of squares was used to assess the percentage variance explained by the country of origin for each of the four animal species. PERMANOVA tests showed that the country of origin explained 3.9% of variance in cattle AGF communities (F test p-value=0.002), 10.2% of variances in horses AGF communities (F test p-value=0.012), 20.62% of variances in goats AGF communities (F test p- value=0.001), and 33.84% of variances in sheep AGF communities (F test p-value=0.001). On the other hand, age explained 6-18% of variances, and sex explained

3.1-18% of variances in community structure.

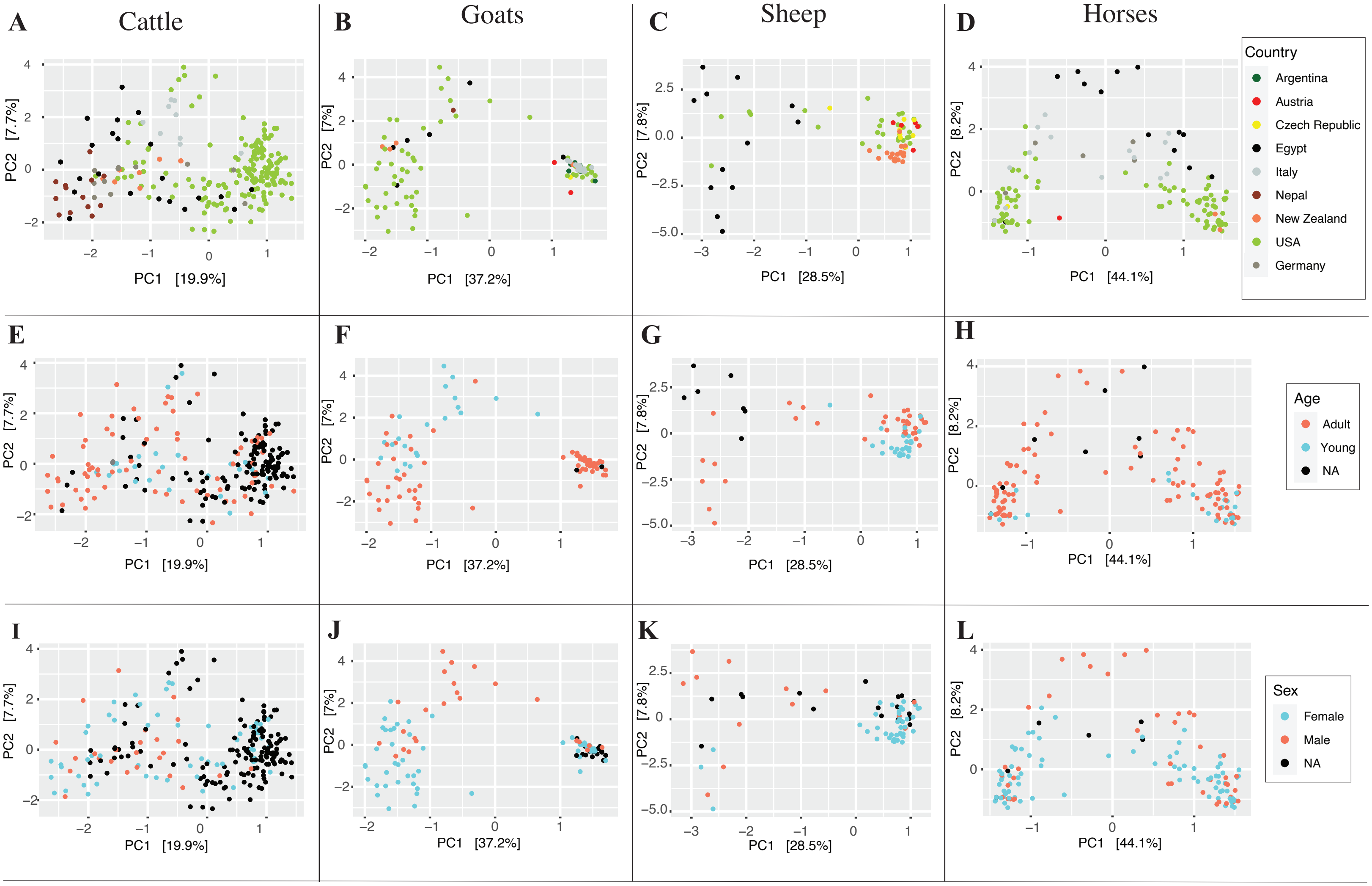

M

Animal species	Factors tested					
	Biogeography		Age		Sex	
	p-value	R^2	p-value	R^2	p-value	R^2
Cattle	0.015	0.038	0.001	0.092	0.001	0.031
Goats	0.001	0.21	0.006	0.06	0.001	0.14
Sheep	0.001	0.34	0.001	0.18	0.001	0.18
Horses	0.001	0.102	0.001	0.094	0.002	0.065

Figure S12. Results of read classification at the phylum level using Kaiju and GOTTCHA2. Only one sample (from a horse) had reads assigned to Neocallimastigomycota with 0.05% total abundance.

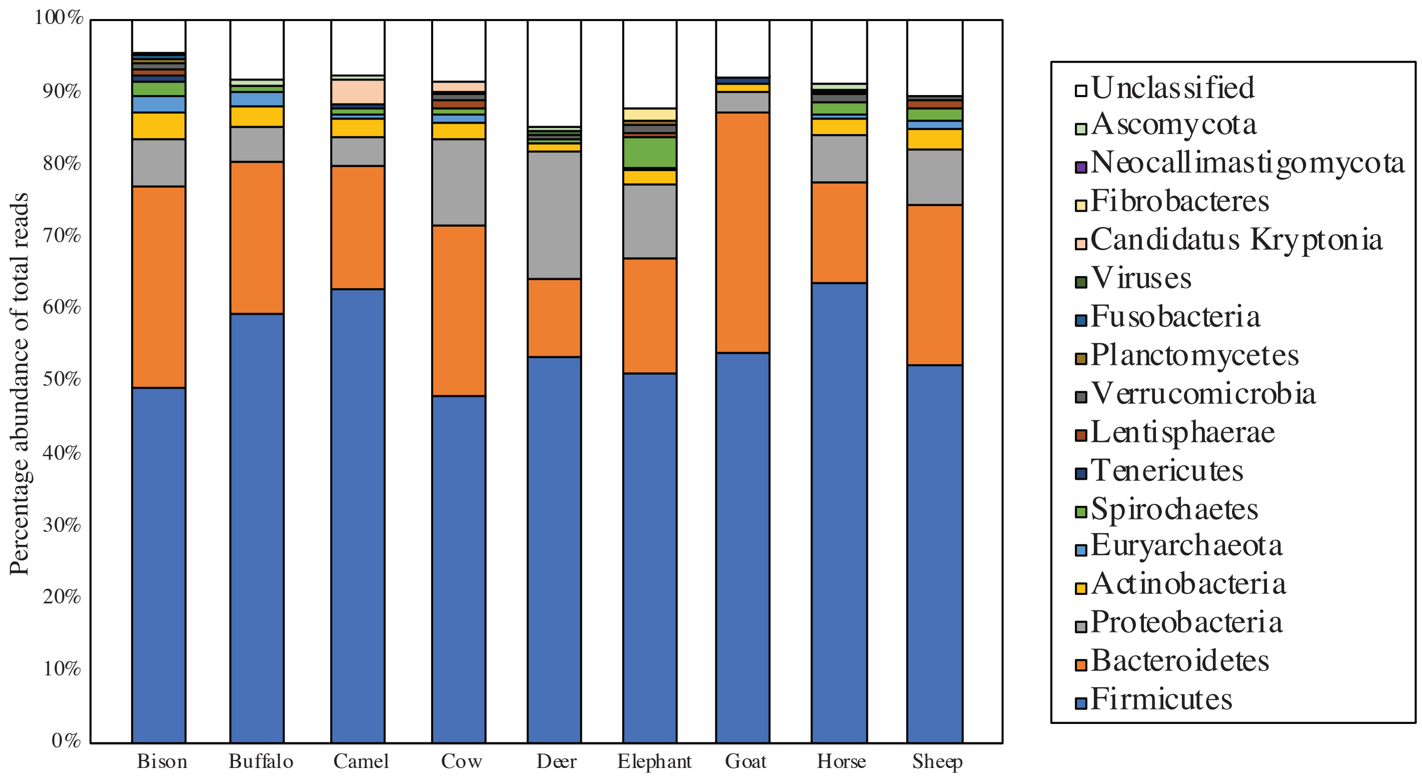

100%
90%
80%
70%
60%
50%
40%
30%
20%
10%
0%
Percentage abundance of total reads
Bison
Buffalo
Camel
Cow
Deer
Elephant
Goat
Horse
Sheep
Unclassified
Ascomycota
Neocallimastigomycota
Fibrobacteres
Candidatus Kryptonia
Viruses
Fusobacteria
Planctomycetes
Verrucomicrobia
Lentisphaerae
Tenericutes
Spirochaetes
Euryarchaeota
Actinobacteria
Proteobacteria
Bacteroidetes
Firmicutes

Figure S13. Quantitative PCR (qPCR) analysis to quantify bacterial:AGF rRNA gene copy number in fecal samples. Values are reported as LSU copies/g fecal sample. Boxplots are showing the distribution of the number of AGF and bacterial rRNA copies/g feces from ten individual cattle, goats, sheep, and horses. Boxplots extend from the first to the third quartile and the median is shown as a thick line in the middle. The whiskers extending on both ends represent variability outside the quartiles and are calculated as follows: Minimum whisker = minimum quartile - 1.5 x inter-quartile range; Maximum whisker = maximum quartile + 1.5 x inter-quartile range. All points outside the box and whiskers are outliers. The number of data points used to calculate each box and whisker plot is n=10.

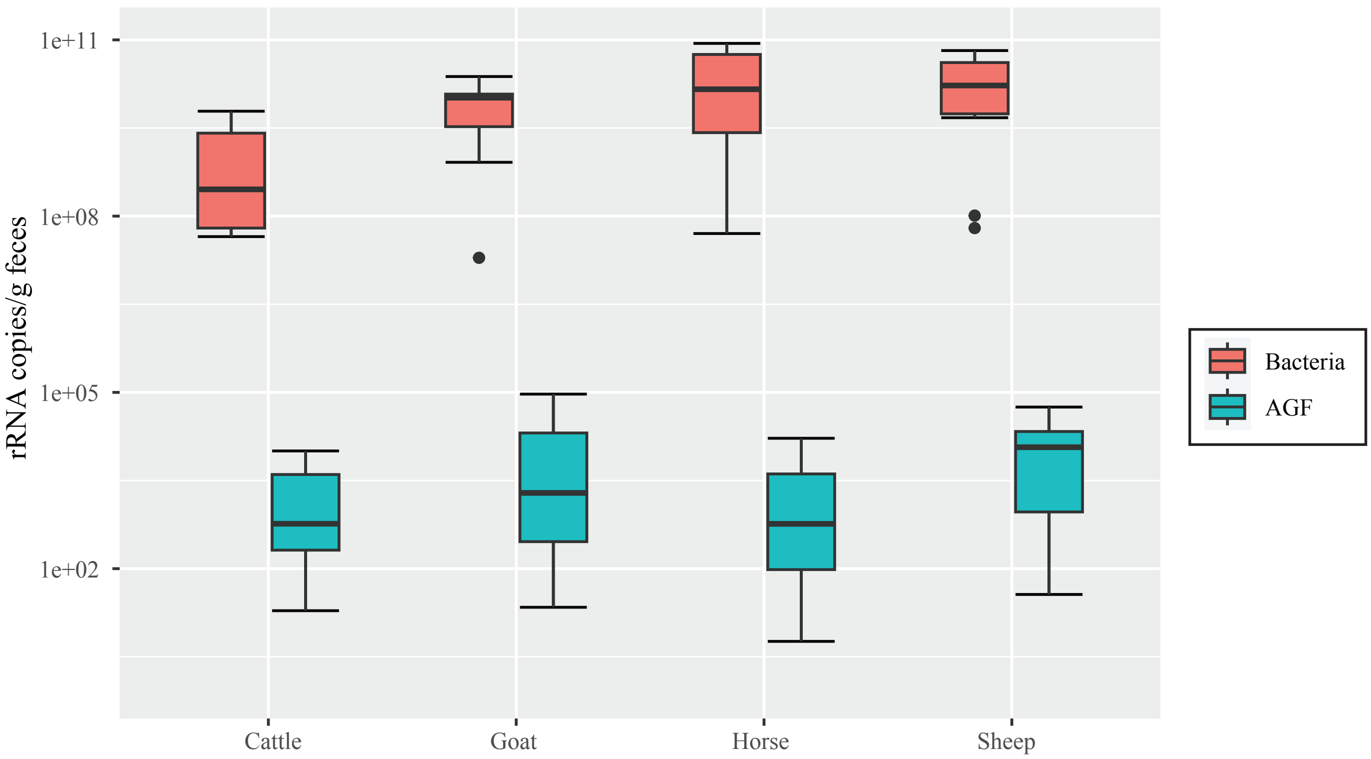

rRNA copies/g feces
1e+11
1e+08
1e+05
1e+02
Cattle
Goat
Horse
Sheep
Bacteria
AGF

Figure S14: Effect of amplicon length/region on pairwise sequence divergence estimates. Comparison of all possible pairwise sequence identities of a group of 206 reference sequences, when the whole D1/D2 region was used (as would be obtained by SMRT sequencing using the NL1 forward primer/ GGNL4 reverse primer, X-axis) and when only the D2 region was used (as would be obtained by Illumina sequencing using primers employed here, Y-axis). Sequences were first aligned in MAFFT, and the alignment was used to calculate pairwise distances in MEGA. Aligned long sequences covering the D1/D2 regions were then trimmed in MEGA to remove the D1 region, and this truncated alignment was then used to calculate pairwise distances in MEGA.

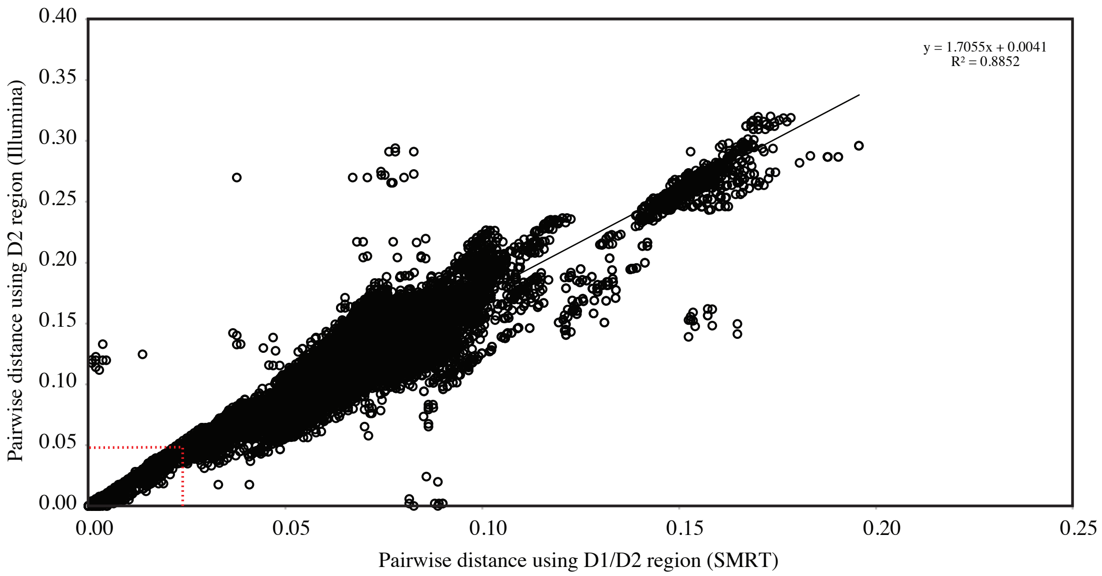

y = 1.7055x + 0.0041
R² = 0.8852
0.40
0.35
0.30
0.25
0.20
0.15
0.10
0.05
0.00
Pairwise distance using D2 region (Illumina)
0.00
0.05
0.10
0.15
0.20
0.25
Pairwise distance using D1/D2 region (SMRT)

Figure S15. Outline of the pipeline used to identify, and bin contigs of potential eukaryotic origin.

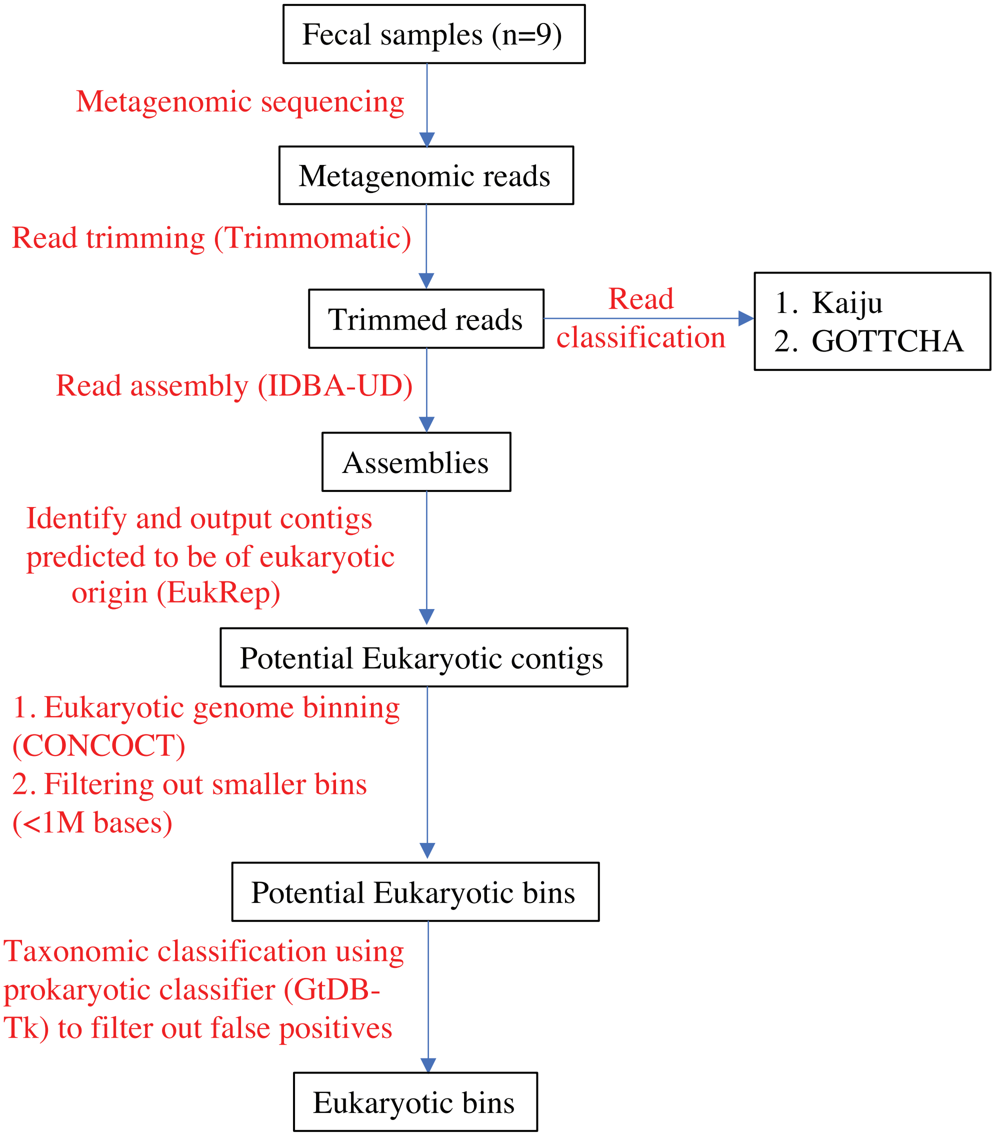
Fecal samples (n=9)
Metagenomic sequencing
Metagenomic reads
Read trimming (Trimmomatic)
Trimmed reads
Read classification
1. Kaiju
2. GOTTCHA
Read assembly (IDBA-UD)
Assemblies
Identify and output contigs predicted to be of eukaryotic origin (EukRep)
Potential Eukaryotic contigs
1. Eukaryotic genome binning (CONCOCT)
2. Filtering out smaller bins (<1M bases)
Potential Eukaryotic bins
Taxonomic classification using prokaryotic classifier (GtDB-Tk) to filter out false positives
Eukaryotic bins

III. Supplementary tables.

Table S1. Summary of previous high throughput culture-independent studies examining AGF diversity in herbivores.

Table S1. Summary of previous high throughput culture-independent studies examining AGF diversity in herbivores.

Reference	Location	Sample type	Sequencing Method	Locus	Total number of sequences	Animals studied							
						Cows (genus Bos)	Horses (including ponies)	Sheep	Goat	Donkey/mule	Deer	Others	Total
**Edwards, et al. 2020a**	Europe, Netherlands, Wageningen	Feces	Illumina	ITS1	~320K					24			24
**Guo, et al. 2020**	Asia, China, Gansu Province	Rumen	Illumina	ITS1	~1.5M			12				6 (Yaks)	18
**Edwards, et al. 2020b**	Europe, Netherlands, Wageningen	Feces	Illumina	ITS1	NA		15			36		13 (zebra)	64
**Kittelmann, et al. 2013**	Oceania, New Zealand, Palmerston North, New Zealand,	Rumen	Pyrosequencing	ITS1	~72K	4		5			2		11
**Kumar, et al. 2015**	North America, United Sattes, PA	Rumen	Pyrosequencing	ITS1	~36K	10							10
**Azad, et al. 2020**	North America, Canada, Lethbridge	Rumen	Illumina	ITS1	~6.6M	12							12
**Fliegerova, et al. 2021**	South America, Argentina, Mendoza City	Rumen	Illumina, Sanger	ITS2, ITS1 (clones), LSU (one sample, clones)	413 ITS1 clones, 35 LSU clones, ~534K Illumina				4				4
**Liggenstoffer, et al. 2010**	North America, United States, Oklahoma City	Feces	Pyrosequencing	ITS1	~270K	1	3	1	1	2	5	17 (multiple animals)	30
**Hanafy, et al. 2020**	North America, United States, Oklahoma and Texas	Feces	SMRT	D1/D2 LSU	~18K	2	1	1	1		4	12 (multiple animals)	21

Edwards JE, Schennink A, Burden F, Long S, van Doorn DA et al. Domesticated equine species and their derived hybrids differ in their fecal microbiota. *Anim Microbiome* 2020a;2:8.
Guo W, Wang W, Bi S, Long R, Ullah F et al. Characterization of Anaerobic Rumen Fungal Community Composition in Yak, Tibetan Sheep and Small Tail Han Sheep Grazing on the Qinghai-Tibetan Plateau. *Animals* 2020;10:144.
Edwards JE, Shetty SA, van den Berg P, Burden F, van Doorn DA et al. Multi-kingdom characterization of the core equine fecal microbiota based on multiple equine (sub)species. *Anim Microbiome* 2020b;2:6.
Kittelmann S, Seedorf H, Walters WA, Clemente JC, Knight R et al. Simultaneous amplicon sequencing to explore co-occurrence patterns of bacterial, archaeal and eukaryotic microorganisms in rumen microbial communities. *PLOS ONE* 2013;8:e47879.
Kumar S, Indugu N, Vecchiarelli B, Pitta DW. Associative patterns among anaerobic fungi, methanogenic archaea, and bacterial communities in response to changes in diet and age in the rumen of dairy cows. *Front Microbiol* , 2015;6:781.
Azad E, Fehr KB, Derakhshani H, Forster R, Acharya S et al. Interrelationships of fiber-associated anaerobic fungi and bacterial communities in the rumen of bloated cattle grazing alfalfa. *Microorganisms* 2020;8:1543.
Fliegerova KO, Podmirseg SM, Vinzelj J, Grilli DJ, Kvasnová S et al. The effect of a high-grain diet on the rumen microbiome of goats with a special focus on anaerobic fungi. *Microorganisms* 2021;9:157.
Liggenstoffer AS, Youssef NH, Couger MB, Elshahed MS. Phylogenetic diversity and community structure of anaerobic gut fungi (phylum Neocallimastigomycota) in ruminant and non-ruminant herbivores. *ISME J* 2010;4:1225-1235.
Hanafy RA, Johnson B, Youssef NH, Elshahed MS. Assessing anaerobic gut fungal diversity in herbivores using D1/D2 large ribosomal subunit sequencing and multi-year isolation. *Environ Microbiol* 2020;22:3883-3908.

Table S2. Results of Wilcoxon test of significance for the distribution of the percentage of novel genera between different animal species (A), animal families (B), animal gut types (C), domestication status (D), and frequency of study (E).

Wilcoxon two-sided, adjusted p-value: ns, not significant (p >0.05); *, 0.01 < p < 0.05; **, p < 0.01; ***, p < 0.001; ****, p < 0.0001.

A

Group1	Group2	p-value	Sig. level	group1	group2	p-value	Sig. level	Group1	Group2	p-value	Sig. level
Alpaca	Horse	0.023	*	Capybara	Goat	0.026	*	Elephant	Goat	1.7E-07	****
	Elephant	0.046	*	Chamois	Elephant	0.038	*		Sheep	7.7E-07	****
Bison	Elephant	0.001	**	Cattle	Goat	3.1E-13	****		Yak	0.003	**
	Deer	0.013	*		Horse	8.9E-11	****		Oryx	0.006	**
	Goat	0.014	*		Elephant	1.2E-06	****		Mara	0.019	*
	Mule	0.018	*		Deer	3.8E-05	****		Manatee	0.038	*
	Camel	0.043	*		Sheep	0.0008	***	Elk	Sheep	0.019	*
Buffalo	Elephant	1.5E-05	****		Mule	0.002	**		Goat	0.024	*
	Horse	0.0005	***		Mara	0.015	*	Giraffe	Sheep	0.026	*
	Mule	0.007	**		Donkey	0.019	*		Goat	0.03	*
	Camel	0.013	*		Elk	0.019	*	Goat	Sheep	8.2E-08	****
	Donkey	0.013	*		Giraffe	0.042	*		Mule	0.002	**
	Elk	0.026	*	Deer	Horse	2.5E-09	****		Mara	0.016	*
	Mara	0.026	*		Elephant	1.7E-08	****		Miniature Zebu	0.018	*
Camel	Deer	1.2E-05	****		Sheep	0.0003	***	Horse	Sheep	4.8E-11	****
	Goat	0.0001	***		Mule	0.002	**		Mara	0.015	*
	Sheep	0.0001	***		Elk	0.006	**		Yak	0.031	*
	Elephant	0.0006	***		Mara	0.006	**		Manatee	0.041	*
	Mule	0.013	*		Miniature Zebu	0.007	**	Mara	Sheep	0.017	*
	Yak	0.024	*		Giraffe	0.011	*	Miniature Zebu	Sheep	0.015	*
	Cattle	0.027	*		Goat	0.029	*	Mule	Sheep	0.001	**
	Elk	0.03	*		Donkey	0.031	*		Yak	0.03	*
	Manatee	0.03	*	Donkey	Goat	0.008	**				
	Mara	0.03	*		Sheep	0.024	*				

B

Group1	Group2	p-value	Sig. level
Bovidae	Equidae	< 2E-16	****
	Elephantidae	3.2E-07	****
	Giraffidae	0.007	**
	Cervidae	0.039	*
Camelidae	Elephantidae	0.0004	***
	Cervidae	0.015	*
	Equidae	0.018	*
	Giraffidae	0.026	*
Cervidae	Equidae	2.1E-08	****
	Elephantidae	1.2E-06	****
	Giraffidae	0.012	*
Elephantidae	Trichechidae	0.038	*
Equidae	Trichechidae	0.042	*

C

Group1	Group2	p-value	Sig. level
Pseudoruminant	Ruminant	0.057	ns
	Hindgut	0.022	*
Ruminant	Hindgut	<2e-16	****

D

Group1	Group2	p-value	Sig. level
Domesticated	Non-domesticated	0.69	ns

E

Group1	Group2	p-value	Sig. level
Rarely/ never studied	Well studied	2.30E-10	****

Table S3. Distribution patterns of novel genera identified in this study*.

Table S3. Distribution patterns of novel genera identified in this study.*

Ubiquity pattern	Relative abundance pattern	Novel genus	No. of samples with abundance <10%	10-25%	25-50%	>50%	Total number of samples
**Ubiquitous** (present in >50% of the samples)	**Frequently abundant** (abundance >10% of the community in >5% of the samples they were encountered)	NY44	445	14	3	4	466
		NY47	351	11	7	3	372
	**Rarely abundant** (abundance >10% of the community in < 5% of the samples they were encountered)	NY9	484	4	1	0	489
		NY42	423	5	0	0	428
		NY1	425	1	0	2	428
		NY20	357	1	1	1	360
		NY53	416	1	0	0	417
		NY15	403	0	1	0	404
		NY11	399	0	0	1	400
		NY10	384	1	0	0	385
		NY17	367	1	0	0	368
	**Always rare** (abundance < 10% in all of the samples they are encountered)	NY7	487	0	0	0	487
		NY8	450	0	0	0	450
		NY6	440	0	0	0	440
		NY4	439	0	0	0	439
		NY19	420	0	0	0	420
		NY28	397	0	0	0	397
		NY52	392	0	0	0	392
		NY21	369	0	0	0	369
		NY23	367	0	0	0	367
		NY22	366	0	0	0	366
		NY55	351	0	0	0	351
		NY5	346	0	0	0	346
		NY24	340	0	0	0	340
		NY2	338	0	0	0	338
		NY16	333	0	0	0	333
		NY35	331	0	0	0	331
**Moderately Ubiquitous** (encountered in 10-50% of samples)	**Rarely abundant** (abundance >10% of the community in < 5% of the samples they were encountered)	NY13	315	1	0	0	316
	**Always rare** (abundance < 10% in all of the samples they are encountered)	NY46	325	0	0	0	325
		NY25	325	0	0	0	325
		NY50	314	0	0	0	314
		NY34	301	0	0	0	301
		NY32	299	0	0	0	299
		NY54	288	0	0	0	288
		NY3	272	0	0	0	272
		NY18	262	0	0	0	262
		NY26	254	0	0	0	254
		NY49	226	0	0	0	226
		NY14	218	0	0	0	218
		NY48	214	0	0	0	214
		NY51	200	0	0	0	200
		NY29	184	0	0	0	184
		NY33	144	0	0	0	144
		NY27	144	0	0	0	144
		NY30	130	0	0	0	130
		NY37	121	0	0	0	121
		NY12	104	0	0	0	104
**Not ubiquitous** (present in <10% of the samples)	**Always rare** (abundance < 10% in all of the samples they are encountered)	NY40	56	0	0	0	56
		NY45	46	0	0	0	46
		NY31	39	0	0	0	39
		NY39	26	0	0	0	26
		NY41	14	0	0	0	14
		NY38	9	0	0	0	9
		NY56	7	0	0	0	7
		NY43	5	0	0	0	5
		NY36	3	0	0	0	3

*Genera were first classified as ubiquitous, moderately ubiquitous, and not ubiquitous. Within these classifications, genera percentage abundance in their respective samples was used to further classify them into frequently abundant, rarely abundant, or always rare. Two novel genera were ubiquitous and frequently abundant, NY47 and NY44, and were both present in high abundance in goats, with occasional high abundance of NY47 in sheep, and occasional high abundance of NY44 in alpaca. Nine genera were ubiquitous but rarely abundant, including NY1, which constituted the absolute majority of the community in the two mara samples, the genera NY15, and NY20 that accounted for 41.8%, and 98.7%, respectively in two rhinoceros' samples, and NY11 that accounted for 60% of the community in one chamois sample. Sixteen novel genera were ubiquitous but always rare. One genus, NY13, was moderately ubiquitous but rarely abundant, where it constituted 11.7% of the community in a single cow sample but made up a minor fraction of the community in all other samples where it was identified (n=315). Nineteen of the 56 novel genera were moderately ubiquitous but always rare, while 8 genera were not ubiquitous and always rare.

Table S4. Topology comparison between trees constructed using Illumina-generated sequences affiliated with novel genera versus SMRT-generated sequences affiliated with novel genera. The novel genera are color coded to reflect congruency between the two trees (yellow, positions congruent; green, positions non-congruent). Blue highlights the 7 novel genera that were missing from the PacBio dataset. These genera exhibited an extremely rare occurrence in the corresponding Illumina-sequenced samples (never exceeding 0.1% in any of the 61 samples) as well as the total dataset (abundances ranging between 0.0004-0.07% in the total Illumina dataset).

Table S4. Topology comparison between trees constructed using Illumina-generated sequences affiliated with novel genera versus SMRT-generated sequences affiliated with novel genera.

Genus	Affiliation/Phylogenetic placement (Illumina tree, Fig 2a)	Affiliation/Phylogenetic placement (SMRT tree, Fig S5)
NY1	Family *Caecomycetaceae*	Basal position
NY2	Family *Piromycetaceae*	Family *Piromycetaceae*
NY3	Basal to the families *Caecomycetaceae* and *Piromycetaceae*	Family *Caecomycetaceae*
NY4	Basal to the families *Caecomycetaceae* and *Piromycetaceae*	Family *Caecomycetaceae*
NY5	Family *Piromycetaceae*	Family *Piromycetaceae*
NY6	Family *Neocallimastigaceae*	Family *Caecomycetaceae*
NY7	Family *Neocallimastigaceae*	Family *Neocallimastigaceae*
NY8	Family *Piromycetaceae*	Family *Piromycetaceae*
NY9	Basal to the families *Caecomycetaceae* and *Piromycetaceae*	Basal to the family *Caecomycetaceae*
NY10	Novel family affiliated with *Buwchfawromyces* and *Tahromyces*	Novel family affiliated with *Buwchfawromyces* and *Tahromyces*
NY11	Novel family affiliated with *Buwchfawromyces* and *Tahromyces*	Novel family affiliated with *Buwchfawromyces* and *Tahromyces*
NY12	Family *Anaeromycetaceae*	Family *Anaeromycetaceae*
NY13	Novel family affiliated with *Joblinomyces*	Novel family affiliated with *Joblinomyces*
NY14	Family *Neocallimastigaceae*	Family *Caecomycetaceae*
NY15	Family *Anaeromycetaceae*	Family *Anaeromycetaceae*
NY16	Family *Anaeromycetaceae*	Family *Anaeromycetaceae*
NY17	Family *Anaeromycetaceae*	Family *Anaeromycetaceae*
NY18	Family *Anaeromycetaceae*	Family *Anaeromycetaceae*
NY19	Family *Anaeromycetaceae*	Family *Anaeromycetaceae*
NY20	Family *Anaeromycetaceae*	Family *Anaeromycetaceae*
NY21	Family *Neocallimastigaceae*	Family *Neocallimastigaceae*
NY22	Family *Neocallimastigaceae*	Family *Neocallimastigaceae*
NY23	Family *Neocallimastigaceae*	Family *Neocallimastigaceae*
NY24	Family *Neocallimastigaceae*	Family *Neocallimastigaceae*
NY25	Family *Caecomycetaceae*	Family *Caecomycetaceae*
NY26	Basal to the families *Caecomycetaceae* and *Piromycetaceae*	Family *Caecomycetaceae*
NY27	Family *Caecomycetaceae*	Family *Caecomycetaceae*
NY28	Family *Neocallimastigaceae*	Family *Neocallimastigaceae*
NY29	Family *Neocallimastigaceae*	Family *Neocallimastigaceae*
NY30	Family *Caecomycetaceae*	Family *Caecomycetaceae*
NY31	Basal to the families *Caecomycetaceae* and *Piromycetaceae*	Missing
NY32	Basal to the families *Caecomycetaceae* and *Piromycetaceae*	Missing
NY33	Family *Caecomycetaceae*	Family *Caecomycetaceae*
NY34	Family *Caecomycetaceae*	Family *Caecomycetaceae*
NY35	Family *Caecomycetaceae*	Family *Caecomycetaceae*
NY36	Novel family affiliated with *Khoyollomyces*	Novel family affiliated with *Khoyollomyces*
NY37	Novel family affiliated with *Aklioshbomyces*	Missing
NY38	Novel family affiliated with *Aklioshbomyces*	Missing
NY39	Novel family affiliated with *Aklioshbomyces*	Missing
NY40	Novel family affiliated with *Aklioshbomyces*	Missing
NY41	Novel family affiliated with *Aklioshbomyces*	Missing
NY42	Novel family affiliated with *Buwchfawromyces* and *Tahromyces*	Novel family affiliated with *Buwchfawromyces* and *Tahromyces*
NY43	Novel family affiliated with *Buwchfawromyces* and *Tahromyces*	Novel family affiliated with *Buwchfawromyces* and *Tahromyces*
NY44	Novel family affiliated with *Joblinomyces*	Novel family affiliated with *Joblinomyces*
NY45	Family *Neocallimastigaceae*	Family *Neocallimastigaceae*
NY46	Novel family affiliated with *Joblinomyces*	Novel family affiliated with *Joblinomyces*
NY47	Novel family affiliated with *Joblinomyces*	Novel family affiliated with *Joblinomyces*
NY48	Family *Neocallimastigaceae*	Novel family affiliated with *Joblinomyces*
NY49	Novel family affiliated with *Joblinomyces*	Novel family affiliated with *Joblinomyces*
NY50	Basal to the families *Caecomycetaceae* and *Piromycetaceae*	Family *Anaeromycetaceae*
NY51	Family *Anaeromycetaceae*	Family *Anaeromycetaceae*
NY52	Family *Neocallimastigaceae*	Family *Neocallimastigaceae*
NY53	Family *Neocallimastigaceae*	Family *Neocallimastigaceae*
NY54	Novel family affiliated with *Khoyollomyces*	Novel family affiliated with *Khoyollomyces*
NY55	Family *Anaeromycetaceae*	Family *Anaeromycetaceae*
NY56	Novel family affiliated with *Khoyollomyces*	Novel family affiliated with *Khoyollomyces*

Color key
Positions matching in both trees
Positions variable
Missing from the SMRT tree

Table S5. Values of normalized stochasticity ratios (NST) calculated using the two indices Bray-Curtis and Jaccard.

Table S5. Values of normalized stochasticity ratios (NST) calculated using two indices.

**Factor**		**Group**	**$NST_{Bray-Curtis}$**	**$NST_{Jaccard}$**
Host-associated factors	Gut type	Hindgut	0.56	0.64
		Foregut Pseudoruminant	0.77	0.58
		Foregut ruminant	0.87	0.79
	Animal family	Elephantidae	0.41	0.35
		Equidae	0.54	0.64
		Camelidae	0.78	0.58
		Bovidae	0.87	0.77
		Cervidae	0.90	0.86
	Animal species	Horse	0.52	0.65
		Goat	0.79	0.70
		Cow	0.81	0.79
		Sheep	0.84	0.66
		Deer	0.95	0.83
Non host-associated factors	Domestication status	Domesticated	0.79	0.77
		Non-domesticated	0.76	0.75

Table S6. Results of Wilcoxon test of significance for the distribution of PACo residuals between different animal species (A), animal families (B), and animal gut types (C).

A

Group1	Group2	p.adj	p.signif
Alpaca	Bison	0.011	**
	Buffalo	0.0028	***
	Cattle	0.0011	****
	Deer	8.40E-05	****
	Donkey	0.031	**
	Elephant	0.0016	****
	Goat	0.0014	****
	Horse	0.043	**
	Mule	0.055	**
	Oryx	0.055	**
	Rhinoceros	0.09	*
	Sheep	0.0014	****
	Yak	0.055	**
	Zebra	0.055	**
Bison	Camel	0.0019	***
	Deer	0.14	*
	Donkey	0.021	**
	Elephant	0.00065	****
	Horse	4.00E-04	****
	Mule	0.039	**
	Rhinoceros	0.039	**
	Zebra	0.039	**
Buffalo	Camel	0.00017	****
	Donkey	0.0067	***
	Elephant	2.80E-05	****
	Horse	5.90E-06	****
	Mule	0.013	**
	Rhinoceros	0.013	**
	Zebra	0.013	**
Camel	Cattle	1.10E-05	****
	Deer	8.20E-07	****
	Donkey	0.033	**
	Goat	1.80E-05	****
	Horse	0.012	**
	Mule	0.016	**
	Oryx	0.016	**
	Rhinoceros	0.049	**
	Sheep	2.10E-05	****
	Yak	0.016	**
	Zebra	0.03	**

Group1	Group2	p.adj	p.signif
Cattle	Deer	0.013	**
	Donkey	0.0032	***
	Elephant	6.30E-07	****
	Horse	1.10E-23	****
	Mule	0.0091	***
	Rhinoceros	0.0091	***
	Zebra	0.0091	***
White-tail deer	Donkey	0.00039	****
	Elephant	3.90E-08	****
	Goat	0.0082	***
	Horse	2.40E-11	****
	Mule	0.0016	****
	Rhinoceros	0.0016	****
	Sheep	0.0039	***
	Yak	0.049	**
	Zebra	0.0016	****
Donkey	Elephant	0.0037	***
	Goat	0.0037	***
	Oryx	0.089	*
	Sheep	0.0038	***
	Yak	0.089	*
Elephant	Mule	0.0091	***
	Oryx	0.0091	***
	Rhinoceros	0.0091	***
	Yak	0.0091	***
	Zebra	0.0091	***
Goat	Elephant	9.90E-07	****
	Mule	0.0091	***
	Rhinoceros	0.0091	***
	Zebra	0.0091	***
Horse	Elephant	9.90E-07	****
	Goat	1.60E-20	****
	Oryx	0.0091	***
	Yak	0.0091	***

B

Group1	Group2	p.adj	p.signif
Camelidae	Bovidae	5.10E-11	****
	Caviidae	0.00021	****
	Cervidae	2.20E-11	****
	Equidae	2.30E-05	****
	Elephantidae	7.50E-08	****
	Giraffidae	0.0033	**
	Trichechidae	0.017	*
	Rhinocerotidae	0.0042	**
Bovidae	Caviidae	0.00034	***
	Cervidae	0.00011	****
	Equidae	3.20E-40	****
	Elephantidae	7.60E-09	****
	Giraffidae	0.0051	**
	Trichechidae	0.021	*
	Rhinocerotidae	0.0013	***

Group1	Group2	p.adj	p.signif
Cervidae	Equidae	6.20E-14	****
	Elephantidae	8.20E-10	****
	Giraffidae	0.001	***
	Trichechidae	0.0069	**
	Rhinocerotidae	0.00017	****
Equidae	Elephantidae	4.70E-08	****
	Giraffidae	0.0056	**
	Trichechidae	0.022	*
Elephantidae	Giraffidae	0.0056	**
	Rhinocerotidae	0.0017	***
Caviidae	Cervidae	3.00E-05	****
	Equidae	0.00053	***
	Elephantidae	0.033	*
	Giraffidae	0.043	*
	Rhinocerotidae	0.021	*

C

Group1	Group2	p.adj	p.signif
Pseudoruminant	Ruminant	7.00E-12	****
	Hindgut	4.40E-07	****
Ruminant	Hindgut	3.50E-51	****

Wilcoxon two-sided, adjusted p-value: *, 0.01 < p < 0.05; **, p < 0.01; ***, p < 0.001;, ****, p < 0.0001.
All pairwise comparisons not shown were not significant (p>0.05)

Table S7. Values of three global phylogenetic signal statistics and their associated p-values for the 37 AGF genera with significant correlations to the host phylogenetic tree*.

Table S7. Values of three phylogenetic signal statistics and their associated p-values for the 37 AGF genera with significant correlations to the host phylogenetic tree*.

Genus	Phylogenetic signal statistic			p-values		
	Abouheif's Cmean	Moran's I	Pagel's Lambda	Abouheif's Cmean	Moran's I	Pagel's Lambda
Orpinomyces	**0.21**	**0.21**	**0.60**	0.001	0.001	0.001
Piromyces	0.08	0.09	**0.31**	0.001	0.001	0.001
Khyollomyces	**0.40**	**0.40**	**0.31**	0.001	0.001	0.001
Cyllamyces	**0.17**	**0.16**	**0.26**	0.001	0.001	0.001
Anaeromyces	**0.14**	**0.13**	**0.31**	0.001	0.001	0.001
Caecomyces	0.09	0.09	**0.28**	0.001	0.001	0.001
Neocallimastix	**0.14**	**0.17**	**0.90**	0.001	0.001	0.001
Liebetanzomyces	0.01	0.01	**0.61**	0.034	0.063	0.001
Paucimyces	0.08	0.09	**0.12**	0.001	0.001	0.001
Pecoramyces	0.01	0.02	**0.99**	0.035	0.021	0.001
Joblinomyces	0.07	0.07	**0.30**	0.002	0.007	0.001
Buwchfawromyces	0.01	0.01	**0.10**	0.031	0.038	1.000
Feramyces	0.00	0.00	**0.98**	0.037	0.261	0.001
Oontomyces	0.02	0.02	**0.42**	0.001	0.001	0.001
NY42	0.05	0.05	0.06	0.001	0.003	0.001
NY44	**0.10**	**0.10**	**0.40**	0.001	0.003	0.001
NY1	**0.52**	**0.95**	**1.00**	0.001	0.001	0.001
NY9	0.08	0.07	0.02	0.004	0.003	0.023
NY10	0.01	0.01	0.07	0.125	0.128	0.031
NY19	0.07	0.08	**0.42**	0.001	0.002	0.001
NY53	0.07	0.07	**0.17**	0.001	0.003	0.001
NY15	0.00	0.00	**0.75**	0.090	0.028	0.001
NY47	**0.10**	**0.10**	**0.21**	0.002	0.004	0.001
NY6	0.05	0.05	0.05	0.001	0.003	0.001
NY7	0.04	0.04	0.04	0.002	0.015	0.002
NY13	0.07	0.07	**0.11**	0.002	0.001	0.001
NY11	0.00	-0.01	**0.99**	0.922	0.965	0.001
NY20	0.08	**0.13**	**0.79**	0.001	0.001	0.001
NY17	0.01	0.01	0.00	0.019	0.021	1.000
NY54	0.00	0.01	**0.87**	0.378	0.083	0.001
AL8	**0.24**	**0.26**	**0.61**	0.001	0.001	0.001
AL4_MN4	**0.10**	**0.10**	**0.19**	0.001	0.002	0.001
MN3	0.05	0.05	0.05	0.003	0.003	0.001
AL3	0.01	0.02	**0.41**	0.021	0.007	0.001
RH1	0.08	0.08	**0.12**	0.003	0.003	0.001
SK3	**0.12**	**0.12**	**0.10**	0.001	0.002	0.001
RH2	**0.10**	**0.13**	**0.79**	0.001	0.004	0.001

* Statistic values >0.1 (as an arbitrary cutoff for correlation) are shown in boldface. Significance is shown in red font for p-values <0.05. All genera with p-value < 0.05 with at least one statistic were considered significant.

Table S8. Significant associations of AGF genera with studied animals based on LIPA values*. Note the high number of strong associations with hindgut animals, and the relatively lower number of strong host-AGF associations in ruminants (only in 3/22 animals: NY19 in bison, RH2 in oryx, AL8 in buffalo, NY9, SK3, and *Caecomyces* in yak, and *Neocallimastix* in elk). However, this lack of strong LIPA signal in foregut fermenters is countered by the identification of multiple intermediate and weak cophylogenetic signals (LIPA values 0.2-1; yellow in heatmap) per animal. For example, goats show intermediate and weak LIPA signals with *Joblinomyces*, NY47, and NY44, sheep show intermediate and weak LIPA signals with NY53 and *Orpinomyces*, buffaloes show intermediate and weak LIPA signals with *Cyllamyces*, NY9, NY14, and *Khoyollomyces*, bison show intermediate and weak LIPA signals with *Orpinomyces*, AL8, and RH1, oryx show intermediate LIPA signals with *Caecomyces*, *Buwchfawromyces*, and AL4/MN4, yak show weak LIPA signals with *Anaeromyces*, AL4, and *Orpinomyces*, deer show intermediate LIPA signals with *Orpinomyces*, NY13, and NY53, and elk show intermediate and weak LIPA signals with *Orpinomyces*, *Caecomyces*, *Khoyollomyces*, AL8, AL4, NY53, NY19, NY42, and NY7.

Table S8. Significant associations of AGF genera with studied animals based on LIPA values*.

Gut type	Family	Animal	Strong (LIPA value>1)	Intermediate (LIPA value 0.4-1)	Weak (LIPA value 0.2-0.4)
**Ruminant**	**Giraffidae**	**Giraffe**		*Orpinomyces*	*Khyollomyces, Cyllamyces, Anaeromyces,* NY42, NY19, NY53, AL8, AL4/MN4
	**Bovidae**	**Goat**		*Joblinomyces* , NY47	NY44
		**Takin**			*Pecoramyces*
		**Chamois**		*Anaeromyces* , AL4/MN4	*Caecomyces, Paucimyces* , NY42, NY6, NY7, AL8
		**Sheep**		*Orpinomyces*	NY53
		**Oryx**	RH2	*Caecomyces* , AL4/MN4, *Buwchfawromyces*	*Khyollomyces, Orpinomyces, Anaeromyces, Paucimyces,* NY6
		**Buffalo**	AL8	*Cyllamyces* , NY9, NY14	*Khyollomyces*
		**Bison**	NY19	*Orpinomyces* , AL8	RH1
		**Yak**	*Caecomyces* , NY9, SK3		*Orpinomyces, Anaeromyces,* AL4/MN4
		**Cow**			*Cyllamyces, Anaeromyces,* AL8
		**Miniature Zebu**		*Paucimyces*	
	**Cervidae**	**Deer**		*Orpinomyces* , NY53, NY13	NY6, AL4/MN4
		**Elk**	*Neocallimastix*	AL8	*Khyollomyces, Orpinomyces, Caecomyces,* NY42, NY19, NY53, NY7, AL4/MN4
**Pseudoruminant**	**Camelidae**	**Alpaca**	*Orpinomyces* , NY44		*Khyollomyces, Piromyces, Caecomyces, Neocallimastix, Paucimyces,* NY42, NY6, NY7
		**Camel**	*Neocallimastix, Oontomyces*	*Orpinomyces*	*Liebetanzomyces* , NY42, NY53, AL4/MN4
**Hindgut**	**Caviidae**	**Capybara**		*Orpinomyces* , AL8, AL4/MN4	*Khyollomyces, Cyllamyces, Anaeromyces, Caecomyces,* NY53
		**Mara**	NY1, *Orpinomyces*	*Piromyces, Anaeromyces,* AL8, AL4/MN4	*Khyollomyces, Cyllamyces, Caecomyces, Neocallimastix, Paucimyces,* NY42, NY19, NY53, NY6, NY7
	**Trichechidae**	**Manatee**	*Paucimyces* , NY54	NY6	
	**Elephantidae**	**Elephant**	*Orpinomyces, Piromyces, Caecomyces*	*Anaeromyces* , AL8, AL4/MN4	*Cyllamyces, Paucimyces,* NY42, NY19, NY53, NY6, NY7
	**Equidae**	**Przewalski's Horse**	*Khyollomyces*		*Orpinomyces*
		**Horse**	*Khyollomyces*		*Orpinomyces, Anaeromyces*
		**Mule**	*Caecomyces, Orpinomyces,* AL3	*Anaeromyces,* AL8, AL4/MN4	*Piromyces, Cyllamyces, Neocallimastix, Paucimyces,* NY42, NY19, NY53, NY6,
		**Zebra**	*Khyollomyces*	*Orpinomyces*	*Piromyces, Cyllamyces,* NY42, NY53, NY6, AL8, AL4/MN4
		**Donkey**	*Piromyces*	*Orpinomyces*	*Cyllamyces, Neocallimastix,* NY42, NY53
	**Rhinocerotidae**	**Rhinoceros**	NY20	*Orpinomyces* , AL4/MN4	*Khyollomyces, Piromyces, Cyllamyces, Paucimyces,* NY53, NY15, AL8

* The following animals showed no significant association with any of the AGF genera: Okapi, Gazelle, Lechwe, Markhor, Ibex, Mountain Goat, Pere Davids Deer, Llama

Table S9. Effect of biogeography (country of origin), age, and sex on the AGF alpha diversity in cattle, horses, goats, and sheep. (A) ANOVA results for the effect of biogeography (country of origin), age, and sex on alpha diversity measures (Shannon, observed number of genera, Simpson, and Inverse Simpson diversity indices). Significant p-values (p-value <0.05) are shown in red text. ANOVA showed that the country of origin had a significant effect on alpha diversity in cattle (with all indices, p-value <0.03), horses (with 3 out of 4 indices, p-value <0.04), but not in goats (with 3 out of 4 indices, p-value >0.1), or sheep (with 3 out of 4 indices, p-value >0.05). On the other hand, animal sex largely had no significant effect on alpha diversity (p-value>0.05). Animal age only showed a significant effect on the alpha diversity of horses (with all indices, p-value <0.03), goats (with all indices, p-value <0.01), and sheep (with 2 out of 4 indices, p-value <0.003), but not cattle. Since out of the three non-host associated factors tested, biogeography showed the most significant effect on alpha diversity, we further carried out Tukey HSD tests for pairwise country (B), and US state (C) comparisons for the two animals with the most significant results (cattle and horses). Cattle samples from the USA, and New Zealand were found to be more diverse than cattle from Germany, but no significant difference was observed in alpha diversity of cattle from all other countries. Similarly, horse samples from USA were found to be more diverse than horses from Germany. Within the USA, the state of origin significantly affected the alpha diversity in cattle, and horses. Analysis of variance showed only significantly higher diversity in cattle originating from OK, in comparison to AZ, CT, and FL, and significantly higher diversity in horses originating from OK, in comparison to CT.

A

Animal Genus	ANOVA p-value				Factor
	Observed Genera	Shannon	Simpson	InvSimpson	
Cattle	5.35E-05	0.0007	0.028	0.014	Biogeography
	0.59	0.64	0.81	0.80	Age
	0.29	0.63	0.33	0.32	Sex
Horses	0.074	0.0006	0.0012	0.038	Biogeography
	0.025	0.0026	0.02	0.018	Age
	0.48	0.15	0.09	0.091	Sex
Goats	0.56	0.82	0.78	0.042	Biogeography
	1.5E-05	9.8E-05	0.0065	0.007	Age
	0.026	0.057	0.09	0.095	Sex
Sheep	0.0003	0.057	0.59	0.6	Biogeography
	7E-05	0.0002	0.3	0.32	Age
	0.006	0.0008	0.7	0.73	Sex

B

Animal Genus	Countries compared		HSD Tukey p-value			
			Observed genera	Shannon	Simpson	InvSimpson
Cattle	New Zealand	Germany	0.039	0.022	0.036	0.032
	USA	Germany	0.00003	0.001	0.072	0.053
Horses	USA	Germany	0.12	0.0028	0.004	0.14

C

Animal Genus	USA states compared		HSD Tukey p-value			
			Observed genera	Shannon	Simpson	InvSimpson
Cattle	Oklahoma	Arizona	0	0.00005	0.0076	0.051
	Oklahoma	Connecticut	0.76	0.012	0.0008	0.32
	Oklahoma	Florida	0	0.0002	0.0067	0.12
Horses	Oklahoma	Connecticut	0.48	0.000002	0.0000006	0.03

Table S10. Results of metagenomic sequencing of the 9 selected samples. Shown are the number of reads and total number of bases before and after quality trimming.

Sample name	Animal	Sequencing output		Following quality trimming	
		Number of reads	Total number of bases	Number of reads	Total number of bases
Cow_121	Cattle	83,926,834	25,261,977,034	80,901,766	21,031,553,774
Goat_Lgoat_25_2	Goat	72,673,118	21,874,608,518	68,489,562	17,562,335,438
Sheep_471	Sheep	84,196,164	25,343,045,364	79,923,682	20,659,377,046
Deer_526	Deer	66,695,652	20,075,391,252	65,036,454	15,804,516,149
Elephant_zoo_499	Elephant	73,457,424	22,110,684,624	70,292,190	17,915,763,157
Horse_168	Horse	77,726,102	23,395,556,702	74,814,616	19,119,611,975
Camel_zoo_839	Camel	57,559,998	17,325,559,398	54,912,228	14,055,855,640
Buffalo_Egypt_E76	Buffalo	42,362,582	12,751,137,182	39,129,976	9,782,563,439
Bison_Zoo_597	Bison	80,459,672	24,218,361,272	77,591,290	18,562,869,841

Table S11. Read assembly results using IDBA-UD for the 9 samples sequenced.

Sample name	Animal	Total number of contigs	No. of contigs > 10,000 bp	No. of contigs > 100,000 bp	Largest contig (bp)	Total length (bp)	N50	L50	GC (%)
Cow_121	Cattle	22,543	1512	12	186,390	106,443,704	5,127	4,599	44.09
Goat_Lgoat_25_2	Goat	37,605	6488	108	402,076	288,827,951	12,265	5,020	46.03
Sheep_471	Sheep	26,307	1606	7	229,995	116,917,695	4,589	5,748	43.78
Deer_526	Deer	17,703	1849	7	142,102	101,222,234	7,179	2,888	48.28
Elephant_zoo_499	Elephant	56,833	4620	8	157,691	283,652,401	5,654	12,544	46.29
Horse_168	Horse	32,942	1707	7	165,452	141,971,471	4,455	8,103	46.56
Camel_zoo_839	Camel	24,891	2889	21	263,806	145,931,569	7,834	4,001	42.65
Buffalo_Egypt_E76	Buffalo	7,448	310	2	116,491	30,156,346	4,013	1,918	45.27
Bison_Zoo_597	Bison	28,642	2644	12	171,965	152,314,276	6,266	5,461	44.3

Table S12. Results of eukaryotic contigs identification using EukRep, and binning using CONCOCT. For bins >1M bp, the GC content and the taxonomic classification using the prokaryotic classifier GTDB-Tk are shown. All bins larger than 1M bp were classified as bacterial with a GC content **>**38%.

Sample name	Animal	EukRep contigs	Binned contigs	Total Number of bins	Bins >1M bp		
					Number of bins	GC content	GTDB taxonomy (Class level)
Cow_121	Cattle	3025	2071	58	3	47.1, 47, 39.8	Bacteroidia
Goat_Lgoat_25_2	Goat	2131	1621	23	2	57.3, 39.7	Clostridia, Bacilli
Sheep_471	Sheep	2756	1888	67	4	38.6, 41.3, 42.8, 46.4	3 Bacteroidia, 1 Clostridia
Deer_526	Deer	1793	1226	23	1	52.3	Clostridia
Elephant_zoo_499	Elephant	5443	3972	56	5	43.6, 60.4, 39.4, 36.7, 47.7	2 Bacteroidia, 1 Kiritimatiellae, 2 Clostridia
Horse_168	Horse	3469	2310	51	4	41.6, 56.4, 45.6, 45	3 Bacteroidia, 1 Kiritimatiellae
Camel_zoo_839	Camel	1917	1379	36	1	44.5	Bacteroidia
Buffalo_Egypt_E76	Buffalo	869	529	21	1	42.1	Clostridia
Bison_Zoo_597	Bison	2864	2118	46	3	42.6, 45, 43.8	Bacteroidia, Clostridia, Bacilli

Table S13. Previous metagenomic studies conducted on herbivorous gut microbiome. Only one study (that employed a deep sequencing strategy (>1 Tbp of data), successfully recovered AGF genomic contigs.

Study animal	Reference	NCBI BioProject ID	Notes
Metagenomic sequencing of the GIT of ruminants	52	PRJNA657455, PRJNA657473	This study produced a total of 6.5 terabytes (Tb) of high quality data, and identified 13 bins belonging to AGF from a total of 28,543 metagenome-assembled genomic bins. These AGF bins were fragmented and only represented <1% of their respective reference genome.
Metagenomic sequencing of African cattle rumen	53	PRJEB39057	No AGF reads/ contigs/ bins were identified.
Metagenomic sequencing of bovine rumen	54	PRJEB23561	No AGF reads/ contigs/ bins were identified.
Metagenomic sequencing of the Caprinae gut microbiota	55	PRJCA008889	No AGF reads/ contigs/ bins were identified.
Metagenomic sequencing of the large intestine of domesticated cattle	56	PRJNA681986	No AGF reads/ contigs/ bins were identified.
Metagenomic sequencing of the cow rumen	57	PRJEB21624	No AGF reads/ contigs/ bins were identified.
Metagenomic sequencing of 283 ruminant cattle	58	PRJEB31266 and PRJEB21624.	No AGF reads/ contigs/ bins were identified.
Metagenomic sequencing of camel gut	59	PRJNA746430	No AGF reads/ contigs/ bins were identified.
Metagenomic sequencing of different fractions of the cow rumen	60	PRJEB47520	No AGF reads/ contigs/ bins were identified.
Metagenomic sequencing of samples from six intestinal regions of dairy cows	61	PRJNA723218	No AGF reads/ contigs/ bins were identified.
Metagenomic sequencing of capybara gut microbiome	62	PRJNA563062.	No AGF reads/ contigs/ bins were identified.
Rumen microbiome of cattle in Japan	63	PRJDB13503	No AGF reads/ contigs/ bins were identified.
Metagenomic sequencing of the beef cattle rumen	64	PRJEB10338 and PRJEB31266	No AGF reads/ contigs/ bins were identified.
Metagenomic sequencing of cattle, sheep, moose, deer, and bison gut microbiomes	65	PRJNA627299 and PRJNA627251	No AGF reads/ contigs/ bins were identified.
Metagenomic sequencing of rumen-fistulated moose	66	PRJNA301235	No AGF reads/ contigs/ bins were identified.
Metagenomic sequencing of the dairy cattle rumen	67	PRJNA526070 and PRJNA597489	No AGF reads/ contigs/ bins were identified.
Metagenomic sequencing of Colombian buffalos' gut microbiome	68	PRJNA605425	No AGF reads/ contigs/ bins were identified.

Metagenomic sequencing of the cow, sheep, reindeer and red deer rumen	[69]	PRJEB34458	No AGF reads/ contigs/ bins were identified.
Metagenomic sequencing of cattle rumen	[70]	PRJNA631951	No AGF reads/ contigs/ bins were identified.
Metagenomic sequencing of the yak fecal microbial community	[71]	PRJNA624740	No AGF reads/ contigs/ bins were identified.
Metagenomic sequencing of the equine fecal microbiome	[72]	PRJNA590977	No AGF reads/ contigs/ bins were identified.
Metagenomic Sequencing of sheep cecal microbiota	[73]	PRJNA702231	No AGF reads/ contigs/ bins were identified.
Metagenomic sequencing of the equine gut microbiome	[74]	PRJNA438436	No AGF reads/ contigs/ bins were identified.
Metagenomic sequencing of samples enriched using cow rumen and sugarcane bagasse	[75]	PRJEB30762	No AGF reads/ contigs/ bins were identified.
Metagenomic sequencing of the moose (Alces alces) rumen	[76]	PRJEB12797	No AGF reads/ contigs/ bins were identified.
Metagenomic sequencing of the gastrointestinal microbiome in dairy cattle	[77]	PRJNA723218	No AGF reads/ contigs/ bins were identified.
Metagenomic sequencing of the microbiome of the buffalo digestive tract	[78]	PRJNA656389	No AGF reads/ contigs/ bins were identified.
Metagenomic sequencing of goats' gut microbiome	[79]	PRJNA792486	No AGF reads/ contigs/ bins were identified.
Metagenomic sequencing of the dairy cattle rumen	[80]	PRJNA883555	No AGF reads/ contigs/ bins were identified.

Supplementary references

1. Mahayri TM*, et al.* Host species affects bacterial evenness, but not diversity: Comparison of fecal bacteria of cows and goats offered the same diet. *Animals* **12**, 2011 (2022).

2. Dagar SS, Kumar S, Mudgil P, Singh R, Puniya AK. D1/D2 Domain of Large-Subunit Ribosomal DNA for Differentiation of Orpinomyces spp. *Appl Environ Microbiol* **77**, (2011).

3. Hanafy RA, Johnson B, Youssef NH, Elshahed MS. Assessing anaerobic gut fungal diversity in herbivores using D1/D2 large ribosomal subunit sequencing and multi-year isolation. *Environ Microbiol* **22**, 3883-3908 (2020).

4. Elshahed MS*, et al.* Characterization and rank assignment criteria for the anaerobic fungi (Neocallimastigomycota). *Int J Syst Evol Microbiol* **72**, doi: 10.1099/ijsem.1090.005449. (2022).

5. Hanafy RA, Johnson B, Youssef NH, Elshahed MS. Assessing anaerobic gut fungal diversity in herbivores using D1/D2 large ribosomal subunit sequencing and multi-year isolation. *Environ Microbiol* **22**, 3883-3908 (2020).

6. Edwards JE, Hermes GDA, Kittelmann S, Nijsse B, Smidt H. Assessment of the accuracy of high-throughput sequencing of the ITS1 region of Neocallimastigomycota for community composition analysis. *Front Micorobiol* **10**, 2370 (2019).

7. Young D*, et al.* Simultaneous metabarcoding and quantification of Neocallimastigomycetes from environmental samples: Insights into community composition and novel lineages. *Microorganisms* **10**, 1749 (2022).

8. Nagler M, Podmirseg SM, Griffith GW, Insam H, Ascher-Jenull J. The use of extracellular DNA as a proxy for specific microbial activity. *Appl Microbiol Biotechnol* **102**, 2885-2898 (2018).

9. Schloss PD*, et al.* Introducing mothur: open-source, platform-independent, community-supported software for describing and comparing microbial communities. *Appl Environ Microbiol* **75**, 7537-7541 (2009).

10. Price MN, Dehal PS, Arkin AP. FastTree 2--approximately maximum-likelihood trees for large alignments. *PLoS One* **5**, e9490 (2010).

11. Minh BQ*, et al.* IQ-TREE 2: new models and efficient methods for phylogenetic inference in the genomic era. *Mol Biol Evol* **37**, 1530-1534 (2020).

12. Kalyaanamoorthy S, Minh BQ, Wong TKF, von Haeseler A, Jermiin LS. ModelFinder: fast model selection for accurate phylogenetic estimates. *Nature Methods* **14**, 587-589 (2017).

13. Hoang DT, Chernomor O, von Haeseler A, Minh BQ, Vinh LS. UFBoot2: Improving the ultrafast bootstrap approximation. *Mol Biol Evol* **35**, 518-522 (2018).

14. García-López R*, et al.* OTUs and ASVs produce comparable taxonomic and diversity from shrimp microbiota 16S profiles using tailored abundance filters. *Genes* **12**, 564 (2021).

15. Wolfgang A*, et al.* Understanding the impact of cultivar, seed origin, and substrate on bacterial diversity of the sugar beet rhizosphere and suppression of soil-borne pathogens. *Front Plant Sci* **11**, 560869 (2020).

16. Inkinen J*, et al.* Diverse and active archaea communities occur in non-disinfected drinking water systems–Less activity revealed in disinfected and hot water systems. *Water Res X* **12**, 100101 (2021).

17. de Souza LMD*, et al.* Diversity, distribution and ecology of fungal communities present in Antarctic lake sediments uncovered by DNA metabarcoding. *Sci Rep* **12**, 8407 (2022).

18. Callahan BJ, McMurdie PJ, Holmes SP. Exact sequence variants should replace operational taxonomic units in marker-gene data analysis. *ISME J* **11**, 2639-2643 (2017).

19. Caruso V, Song X, Asquith M, Karstens L. Performance of microbiome sequence inference methods in environments with varying biomass. *mSystems* **4**, e00163-00118 (2019).

20. Fricker AM, Podlesny D, Fricke WF. What is new and relevant for sequencing-based microbiome research? A mini-review. *J Adv Res* **19**, 105-112 (2019).

21. Joos L*, et al.* Daring to be differential: metabarcoding analysis of soil and plant-related microbial communities using amplicon sequence variants and operational taxonomical units. *BMC Genomics* **21**, 733 (2020).

22. Ning D, Deng Y, Tiedje JM, Zhou J. A general framework for quantitatively assessing ecological stochasticity. *Proc Natl Acad Sci USA* **116**, 16892-16898 (2019).

23. Stegen JC, Lin X, Fredrickson JK, Konopka AE. Estimating and mapping ecological processes influencing microbial community assembly. *Front Microbiol* **6**, 370 (2015).

24. Zhou J, Ning D. Stochastic community assembly: Does It matter in microbial ecology? *Microbiol Mol Biol Rev* **81**, e00002-00017 (2017).

25. Anderson MJ, Walsh DCI. PERMANOVA, ANOSIM, and the Mantel test in the face of heterogeneous dispersions: What null hypothesis are you testing? *Ecol Monographs* **83**, 557-574 (2013).

26. Youngblut ND*, et al.* Vertebrate host phylogeny influences gut archaeal diversity. *Nat Microbiol* **6**, 1443-1454 (2021).

27. Murphy CL*, et al.* Horizontal gene transfer forged the evolution of anaerobic gut fungi into a phylogenetically distinct gut-dwelling fungal lineage. . *Appl Environ Microbiol* **85**, e00988-00919 (2019).

28. Wang Y, Youssef NH, Couger MB, Hanafy RA, Elshahed MS, Stajich JE. Molecular dating of the emergence of anaerobic rumen fungi and the impact of laterally acquired genes. *mSystems* **4**, e00247-00219 (2019).

29. Hanafy RA*, et al.* Seven new Neocallimastigomycota genera from wild, zoo-housed, and domesticated herbivores greatly expand the taxonomic diversity of the phylum. . *Mycologia 112, :1212-1239 (2020).*

30. Gruninger RJ*, et al.* Application of transcriptomics to compare the carbohydrate active enzymes that are expressed by diverse genera of anaerobic fungi to degrade plant cell wall carbohydrates. *Front Microbiol* **9**, 1581 (2018).

31. Haitjema CH*, et al.* A parts list for fungal cellulosomes revealed by comparative genomics. *Nature Microbiol* **2**, 17087 (2017).

32. Wilken SE*, et al.* Experimentally validated reconstruction and analysis of a genome-scale metabolic model of an anaerobic Neocallimastigomycota fungus. *mSystems* **16**, e00002-00021 (2021).

33. Li Y*, et al.* Combined genomic, transcriptomic, proteomic, and physiological characterization of the growth of *Pecoramyces* sp. F1 in monoculture and co-culture with a syntrophic methanogen. *Front Microbiol* **10**, 435 (2019).

34. Calkins S, Elledge NC, Hanafy RA, Elshahed MS, Youssef N. A fast and reliable procedure for spore collection from anaerobic fungi: Application for RNA uptake and long-term storage of isolates. *J Microbiol Meth* **127**, 206-213 (2016).

35. Fu L, Niu B, Zhu Z, Wu S, Li W. CD-HIT: accelerated for clustering the next-generation sequencing data. *Bioinformatics* **28**, 3150-3152 (2012).

36. Manni M, Berkeley MR, Seppey M, Simão FA, Zdobnov EM. BUSCO Update: Novel and Streamlined Workflows along with Broader and Deeper Phylogenetic Coverage for Scoring of Eukaryotic, Prokaryotic, and Viral Genomes. *Mol Biol Evol* **38**, 4647-4654 (2021).

37. Hanafy RA, Wang Y, Stajich JE, Pratt CJ, Youssef NH, Elshahed MH. Phylogenomic analysis of the Neocallimastigomycota: proposal of *Caecomycetaceae* fam. nov., *Piromycetaceae* fam. nov., and emended description of the families *Neocallimastigaceae* and *Anaeromycetaceae*. *Int J Syst Evol Microbiol* **273**, https://doi.org/10.1099/ijsem.1090.005735 (2023).

38. Capella-Gutiérrez S, Silla-Martínez JM, Gabaldón T. trimAl: a tool for automated alignment trimming in large-scale phylogenetic analyses. *Bioinformatics* **25**, 1972-1973 (2009).

39. Lanfear R, Frandsen PB, Wright AM, Senfeld T, Calcott B. PartitionFinder 2: new methods for selecting partitioned models of evolution for molecular and morphological phylogenetic analyses. *Mol Biol Evol* **34**, 772-773 (2016).

40. Suchard MA, Lemey P, Baele G, Ayres DL, Drummond AJ, Rambaut A. Bayesian phylogenetic and phylodynamic data integration using BEAST 1.10. *Virus Evol* **4**, vey016 (2018).

41. Rambaut A, Drummond AJ, Xie D, Baele G, Suchard MA. Posterior summarization in Bayesian phylogenetics using Tracer 1.7. *Syst Biol* **67**, 901-904 (2018).

42. Bolger AM, Lohse M, Usadel B. Trimmomatic: a flexible trimmer for Illumina sequence data. *Bioinformatics* **30**, 2114-2120 (2014).

43. Menzel P, Ng KL, Krogh A. Fast and sensitive taxonomic classification for metagenomics with Kaiju. *Nat Commun* **7**, 11257 (2016).

44. Peng Y, Leung HC, Yiu SM, Chin FY. IDBA-UD: a de novo assembler for single-cell and metagenomic sequencing data with highly uneven depth. *Bioinformatics* **28**, 1420-1428 (2012).

45. West PT, Probst AJ, Grigoriev IV, Thomas BC, Banfield JF. Genome-reconstruction for eukaryotes from complex natural microbial communities. *Genome Res* **28**, 569-580 (2018).

46. Alneberg J*, et al.* Binning metagenomic contigs by coverage and composition. *Nature Methods* **11**, 1144-1146 (2014).

47. Chaumeil P-A, Mussig AJ, Hugenholtz P, Parks DH. GTDB-Tk v2: memory friendly classification with the genome taxonomy database. *Bioinformatics* **38**, 5315-5316 (2022).

48. Allen B, Drake M, Harris N, Sullivan T. Using KBase to assemble and annotate prokaryotic genomes. *Curr Protoc Microbiol* **46**, 1e.13.11-11e.13.18 (2017).

49. Arkin AP*, et al.* KBase: The United States Department of Energy systems biology knowledgebase. *Nat Biotechnol* **36**, 566-569 (2018).

50. Chivian D*, et al.* Metagenome-assembled genome extraction and analysis from microbiomes using KBase. *Nat Protoc* **18**, 208-238 (2023).

51. Wang Y, Qian PY. Conservative fragments in bacterial 16S rRNA genes and primer design for 16S ribosomal DNA amplicons in metagenomic studies. *PLoS One* **4**, e7401 (2009).

52. Xie F*, et al.* An integrated gene catalog and over 10,000 metagenome-assembled genomes from the gastrointestinal microbiome of ruminants. *Microbiome* **9**, 137 (2021).

53. Wilkinson T*, et al.* 1200 high-quality metagenome-assembled genomes from the rumen of African cattle and their relevance in the context of sub-optimal feeding. *Genome Biol* **21**, 229 (2020).

54. Li J*, et al.* A catalog of microbial genes from the bovine rumen unveils a specialized and diverse biomass-degrading environment. *Gigascience* **9**, (2020).

55. Zhang XX*, et al.* A catalog of over 5,000 metagenome-assembled microbial genomes from the Caprinae gut microbiota. *Microbiol Spectr* **10**, e0221122 (2022).

56. Teseo S*, et al.* A global phylogenomic and metabolic reconstruction of the large intestine bacterial community of domesticated cattle. *Microbiome* **10**, 155 (2022).

57. Stewart RD*, et al.* Assembly of 913 microbial genomes from metagenomic sequencing of the cow rumen. *Nat Commun* **9**, 870 (2018).

58. Stewart RD, Auffret MD, Warr A, Walker AW, Roehe R, Watson M. Compendium of 4,941 rumen metagenome-assembled genomes for rumen microbiome biology and enzyme discovery. *Nat Biotechnol* **37**, 953-961 (2019).

59. Gharechahi J, Sarikhan S, Han JL, Ding XZ, Salekdeh GH. Functional and phylogenetic analyses of camel rumen microbiota associated with different lignocellulosic substrates. *NPJ Biofilms Microbiomes* **8**, 46 (2022).

60. Hernández R, Chaib De Mares M, Jimenez H, Reyes A, Caro-Quintero A. Functional and phylogenetic characterization of bacteria in bovine rumen using fractionation of ruminal fluid. *Front Microbiol* **13**, 813002 (2022).

61. Lin L*, et al.* Genome-centric investigation of bile acid metabolizing microbiota of dairy cows and associated diet-induced functional implications. *ISME J* **17**, 172-184 (2023).

62. Cabral L*, et al.* Gut microbiome of the largest living rodent harbors unprecedented enzymatic systems to degrade plant polysaccharides. *Nat Commun* **13**, 629 (2022).

63. Sato Y*, et al.* Identification of 146 Metagenome-assembled Genomes from the Rumen Microbiome of Cattle in Japan. *Microbes Environ* **37**, (2022).

64. Auffret MD, Stewart RD, Dewhurst RJ, Duthie CA, Watson M, Roehe R. Identification of microbial genetic capacities and potential mechanisms within the rumen microbiome explaining differences in beef cattle feed efficiency. *Front Microbiol* **11**, 1229 (2020).

65. Anderson CL, Fernando SC. Insights into rumen microbial biosynthetic gene cluster diversity through genome-resolved metagenomics. *Commun Biol* **4**, 818 (2021).

66. Solden LM*, et al.* Interspecies cross-feeding orchestrates carbon degradation in the rumen ecosystem. *Nat Microbiol* **3**, 1274-1284 (2018).

67. Xue MY*, et al.* Investigation of fiber utilization in the rumen of dairy cows based on metagenome-assembled genomes and single-cell RNA sequencing. *Microbiome* **10**, 11 (2022).

68. Aguilar-Marin SB, Betancur-Murillo CL, Isaza GA, Mesa H, Jovel J. Lower methane emissions were associated with higher abundance of ruminal Prevotella in a cohort of Colombian buffalos. *BMC Microbiol* **20**, 364 (2020).

69. Glendinning L, Genç B, Wallace RJ, Watson M. Metagenomic analysis of the cow, sheep, reindeer and red deer rumen. *Sci Rep* **11**, 1990 (2021).

70. Gharechahi J, Vahidi MF, Bahram M, Han JL, Ding XZ, Salekdeh GH. Metagenomic analysis reveals a dynamic microbiome with diversified adaptive functions to utilize high lignocellulosic forages in the cattle rumen. *ISME J* **15**, 1108-1120 (2021).

71. Gong G, Zhou S, Luo R, Gesang Z, Suolang S. Metagenomic insights into the diversity of carbohydrate-degrading enzymes in the yak fecal microbial community. *BMC Microbiol* **20**, 302 (2020).

72. Gilroy R*, et al.* Metagenomic investigation of the equine faecal microbiome reveals extensive taxonomic diversity. *PeerJ* **10**, e13084 (2022).

73. Xie F, Xu L, Wang Y, Mao S. Metagenomic sequencing reveals that high-grain feeding alters the composition and metabolism of cecal microbiota and induces cecal mucosal injury in sheep. *mSystems* **6**, e0091521 (2021).

74. Mach N*, et al.* Mining the equine gut metagenome: poorly-characterized taxa associated with cardiovascular fitness in endurance athletes. *Commun Biol* **5**, 1032 (2022).

75. Tomazetto G, Pimentel AC, Wibberg D, Dixon N, Squina FM. Multi-omic directed discovery of cellulosomes, polysaccharide utilization loci, and lignocellulases from an enriched rumen anaerobic consortium. *Appl Environ Microbiol* **86**, (2020).

76. Svartström O*, et al.* Ninety-nine de novo assembled genomes from the moose (Alces alces) rumen microbiome provide new insights into microbial plant biomass degradation. *ISME J* **11**, 2538-2551 (2017).

77. Lin L, Lai Z, Zhang J, Zhu W, Mao S. The gastrointestinal microbiome in dairy cattle is constrained by the deterministic driver of the region and the modified effect of diet. *Microbiome* **11**, 10 (2023).

78. Tong F*, et al.* The microbiome of the buffalo digestive tract. *Nat Commun* **13**, 823 (2022).

79. Zhang T*, et al.* Transmission of the gut microbiome in cohousing goats and pigs. *Front Microbiol* **13**, 948617 (2022).

80. Zhang L, Shen H, Zhang J, Mao S. Variety of rumen microbial populations involved in biohydrogenation related to individual milk fat percentage of dairy cows. *Front Vet Sci* **10**, 1106834 (2023).
